# Comprehensive Insights Into Forensic Features and Genetic Background of Chinese Northwest Hui Group Using Six Distinct Categories of 231 Molecular Markers

**DOI:** 10.3389/fgene.2021.705753

**Published:** 2021-10-15

**Authors:** Chong Chen, Xiaoye Jin, Xingru Zhang, Wenqing Zhang, Yuxin Guo, Ruiyang Tao, Anqi Chen, Qiannan Xu, Min Li, Yue Yang, Bofeng Zhu

**Affiliations:** ^1^ Key Laboratory of Shaanxi Province for Craniofacial Precision Medicine Research, College of Stomatology, Xi’an Jiaotong University, Xi’an, China; ^2^ Guangzhou Key Laboratory of Forensic Multi-Omics for Precision Identification, School of Forensic Medicine, Southern Medical University, Guangzhou, China; ^3^ Shanghai Key Laboratory of Forensic Medicine, Shanghai Forensic Service Platform, Academy of Forensic Sciences, Ministry of Justice, Shanghai, China; ^4^ Department of Forensic Medicine, Shanghai Medical College of Fudan University, Shanghai, China; ^5^ Institute of Forensic Medicine, West China School of Basic Medical Sciences and Forensic Medicine, Sichuan University, Chengdu, China; ^6^ School of Basic Medicine, Inner Mongolia Medical University, Hohhot, China; ^7^ Department of Forensic Genetics, Multi-Omics Innovative Research Center of Forensic Identification, School of Forensic Medicine, Southern Medical University, Guangzhou, China

**Keywords:** Chinese Hui minority, population genentics, forensics, molecular markers, MPS

## Abstract

The Hui minority is predominantly composed of Chinese-speaking Islamic adherents distributed throughout China, of which the individuals are mainly concentrated in Northwest China. In the present study, we employed the length and sequence polymorphisms-based typing system of 231 molecular markers, i.e., amelogenin, 22 phenotypic-informative single nucleotide polymorphisms (PISNPs), 94 identity-informative single nucleotide polymorphisms (IISNPs), 24 Y-chromosomal short tandem repeats (Y-STRs), 56 ancestry-informative single nucleotide polymorphisms (AISNPs), 7 X-chromosomal short tandem repeats (X-STRs), and 27 autosomal short tandem repeats (A-STRs), into 90 unrelated male individuals from the Chinese Northwest Hui group to comprehensively explore its forensic characteristics and genetic background. Total of 451 length-based and 652 sequence-based distinct alleles were identified from 58 short tandem repeats (STRs) in 90 unrelated Northwest Hui individuals, denoting that the sequence-based genetic markers could pronouncedly provide more genetic information than length-based markers. The forensic characteristics and efficiencies of STRs and IISNPs were estimated, both of which externalized high polymorphisms in the Northwest Hui group and could be further utilized in forensic investigations. No significant departure from the Hardy–Weinberg equilibrium (HWE) expectation was observed after the Bonferroni correction. Additionally, four group sets of reference population data were exploited to dissect the genetic background of the Northwest Hui group separately from different perspectives, which contained 26 populations for 93 IISNPs, 58 populations for 17 Y-STRs, 26 populations for 55 AISNPs (raw data), and 109 populations for 55 AISNPs (allele frequencies). As a result, the analyses based on the Y-STRs indicated that the Northwest Hui group primarily exhibited intimate genetic relationships with reference Hui groups from Chinese different regions except for the Sichuan Hui group and secondarily displayed close genetic relationships with populations from Central and West Asia, as well as several Chinese groups. However, the AISNP analyses demonstrated that the Northwest Hui group shared more intimate relationships with current East Asian populations apart from reference Hui group, harboring the large proportion of ancestral component contributed by East Asia.

## Introduction

For recent years, the extensive applications of DNA analysis technologies have made it a crucial tool in forensic investigations. To date, the genomic DNA from biological samples is predominantly dissected by PCR and capillary electrophoresis (CE)-based method to reveal the length variations in genetic markers, such as short tandem repeats (STRs). Further, the DNA sequencing technology also serves as an important role in providing comprehensive information of the target DNA in forensic applications. Conventional Sanger sequencing, initially introduced in the 1970s, has enabled enormous progress in the fields of molecular biology, genomics, and genetics and been regarded as the standard sequencing technology in the forensic investigations (Sanger et al., 1977). Yet, the shortcomings of Sanger sequencing technology, such as low throughput and sensitivity, have hampered its utilization in the more in-depth and intricate genome analyses, which facilitate the exploration of other high-throughput DNA technologies in forensic studies (Fullwood et al., 2009). Massively parallel sequencing (MPS), or next-generation sequencing (NGS), has become an emerging tool commonly utilized in forensic genetic fields (Børsting and Morling, 2015; Li et al., 2017). MPS technology has advantages such as simultaneous sequencing of multiple types of genetic molecular markers and detecting samples at an extraordinarily high throughput capacity, which make it possible to yield forensic data containing more information in a single reaction (England et al., 2020). In addition, the polymorphisms of sequence variations contained in different genetic molecular markers which are undetectable by traditional CE technology, like STRs, are easily identified using MPS technology platforms, thus increasing the possibility of discovering new STR alleles (Gettings et al., 2015; Churchill et al., 2016; Novroski et al., 2016). MPS platforms also maintain the conventional abilities to identify STRs length polymorphisms, facilitating the compatibility of currently forensic DNA data generated by PCR and CE-based platform (Parson et al., 2016; Bruijns et al., 2018).

The ForenSeq™ DNA Signature Prep Kit (Verogen Inc., San Diego, CA, United States), a newly developed MPS-based commercial kit, is a length and sequence polymorphisms-based typing system for three kinds of STRs and three types of single nucleotide polymorphisms (SNPs), which can simultaneously detect 231 genetic molecular markers in a single reaction on MiSeq FGx™ Forensic Genomics System (Verogen Inc., San Diego, CA, United States). This kit provides two different primer mixes, including primer mixes A and B. Primer mix A is designed to detect amelogenin, 27 autosomal STRs (A-STRs), 24 Y-chromosomal STRs (Y-STRs), 7 X-chromosomal STRs (X-STRs), and 94 identity informative SNPs (IISNPs); and primer mix B contains primer mix A plus the primers for 22 phenotypic informative SNPs (PISNPs) and 56 biogeographical ancestry informative SNPs (AISNPs) (Jäger et al., 2017). To date, the system performance of the ForenSeq™ DNA Signature Prep Kit has been comprehensively evaluated by investigating the reproducibility, sensitivity, concordance, casework-type sample, and inter-laboratory comparison and validation, which has proven it to be a promising tool in forensic applications over recent years (Just et al., 2017; Xavier and Parson, 2017; Köcher et al., 2018). Further, several studies have demonstrated that this kit is suitable for challenging samples such as forensically degraded and mixed samples from crime scenes (Fattorini et al., 2017; Sharma et al., 2020), and also performs well in the kinship analyses (Li et al., 2018), phenotypic and biogeographical ancestry predictions (Sharma et al., 2019), and in the exploratory studies of population genetics (Delest et al., 2020).

The Chinese Hui ethnic group is one of the national minorities officially recognized by the People’s Republic of China and widespread throughout China, including 34 provincial-level administrative regions. According to the population distribution, the Chinese Hui group is more concentrated in Northwest China. Intriguingly, the Hui group occupies a special existence among the Chinese ethnic minorities, of which the individuals are portrayed as Muslims who appear culturally and linguistically similar to the Han population. Currently, population genetic studies concentrated on limited kinds of genetic markers have been conducted on Hui groups from Chinese different regions. For example, Zou et al. detected the genetic polymorphisms of 30 insertion/deletion (InDels) in the Guangxi Hui group (Zou et al., 2020). HLA class I polymorphisms were investigated in the Hui group from Chinese Qinghai province by Hong et al. (2007). The genomic makeup and ancestry background of the Hui group from Sichuan province were explored using over 700K SNPs by Liu et al. (2021). Guo et al. investigated the population structure of the Hui group from Chinese Liaoning province based on 17 Y-STR loci (Guo, 2017). Yao et al. and Liu et al. explored the genetic background of the Hui group from Chinese Gansu province using 15 autosomal STRs (Yao et al., 2016) and 27 Y-STRs (Liu et al., 2018) loci, respectively. The genetic polymorphisms of the Hui group from Chinese Ningxia Hui (NXH) autonomous region were estimated by various research teams using 15 STRs (Ma et al., 2017), 24 Y-STRs (Zhu et al., 2014), 30 InDels (Zhou et al., 2020), 12 X-STRs (Meng et al., 2014), and 17 Y-STR loci (Guo et al., 2008), respectively. Thirty InDels (Xie et al., 2018), 30 AISNPs (Jin et al., 2020), 22 STRs (Fang et al., 2018), and 39 ancestry-informative marker (AIM) InDel loci (Xie et al., 2020) were also separately applied in other Hui groups from Northwest China.

In fact, different opinions on the genetic origin and ancestral history of the Chinese Hui group have existed for years. Two kinds of hypotheses have been proposed to infer the historical formation of the Chinese Hui group, which are demic diffusion and cultural diffusion hypotheses (Liu et al., 2021). In addition, due to the limited types of genetic markers utilized separately in previous studies mentioned above, it might be more necessary to simultaneously enroll various types of genetic markers to provide more extensive information for exploring the genetic structure and ancestral origin of the Chinese Hui group. Thus, in the present study, we implemented 231 genetic markers (amelogenin, 27 A-STRs, 24 Y-STRs, 7 X-STRs, 94 IISNPs, 22 PISNPs, and 56 AISNPs) from the ForenSeq™ DNA Signature Prep Kit into the Hui group from Northwest China. Both length- and sequence-based polymorphisms of genetic markers were utilized into 90 unrelated male individuals recruited from the Northwest Hui group, with the intention of comprehensively detecting the forensic characteristics and genetic background of the Northwest Hui group and subsequently enriching the genetic information of the Chinese populations.

## Materials and Methods

### Sample Information

In total, 90 blood samples from unrelated healthy Hui male individuals from Northwest China were collected. All the participants provided their written informed consents prior to sample collection. The present research was conducted according to the ethical guidelines of the Xi’an Jiaotong University Health Science Center and further authorized by the Ethical committees of the Xi’an Jiaotong University Health Science Center (approval number: 2019-1039; 2020-1382).

### Library Preparation

Library preparation was performed in accordance with the recommendation of the ForenSeq™ DNA Signature Prep Kit (Illumina Inc., CA, United States) (Illumina, 2015). The first stage was to amplify and tag the DNA targets. One disc containing a 1.2 mm-diameter blood sample was directly amplified without DNA extraction in the ForenSeq™ Sample Plate. PCR was conducted on the GeneAmp PCR System 9700 Thermal Cycler (Applied Biosystems, CA, United States) according to the following parameters: 98°C for 3 min; eight cycles of 96°C for 45 s, 80°C for 30 s, 54°C for 2 min, and 68°C for 2 min; 10 cycles of 96°C for 30 s, 68°C for 3 min; 68°C for 10 min; and hold at 10°C. The first-round PCR products were subsequently utilized in the second-stage PCR to enrich the targets. The index adapters and sequences required for cluster amplification were also added in the second-stage PCR. The detailed PCR parameters were as follows: 98°C for 30 s; 15 cycles of 98°C for 20 s, 66°C for 30 s, and 68°C for 90 s; 68°C for 10 min; and hold at 10°C. All samples were amplified with DNA primer mix B. The amplified DNA libraries were then purified from the remaining reaction components using purification beads, and the concentrations of the libraries were normalized to ensure a consistent cluster density. The normalized libraries required to sequence on the same flow cell were then mixed in equal volumes during the library pooling stage. The mixed libraries were then diluted in a hybridization buffer, added the human sequencing control and denatured at 96°C in preparation for sequencing. Finally, after denaturation and dilution, the mixed libraries were sequenced on the MiSeq Desktop Sequencer using the MiSeq FGx Forensic Genomics System (Illumina Inc., CA, United States) (Illumina, 2015).

### Data Analyses and Interpretation

The generated data were processed using the ForenSeq™ Universal Analysis Software based on the default thresholds. In detail, the analytical threshold (AT), interpretation threshold (IT), stutter filter (SF), and intra-locus balance (IB) were utilized to estimate the data quality (Illumina, 2015; Köcher et al., 2018). The optimized AT values were 1.5% for all loci except for DYS635 (3.3%), DYS389II (5%), and DYS448 (3.3%) loci, which represented the lower boundary for the valid results accounting for the entire read count per locus. The default IT values were 4.5% for all loci apart from DYS635 (10%), DYS389II (15%), and DYS448 (10%) loci, indicating the upper boundary of the uncertainty range. When the resulting values are between the AT and IT, the user should identify whether an actual variant has occurred. The SF values for all STR loci were extended from 7.5% to 50% with the average value as 19%. The general settings of IB values were 60% for STRs and 50% for SNPs. As for the valid sequencing depth, the minimum depths were set as 10× for STRs and 5× for SNPs. For A-STRs, the parameters for both sequence- and length-based polymorphisms were calculated using STRAF software v1.0.5 (Gouy and Zieger, 2017), including observed heterozygosity (H_obs_), expected heterozygosity (H_exp_), polymorphism information content (PIC), power of discrimination (PD), exclusion probability (PE), match probability (PM), typical paternity index (TPI), and the Hardy–Weinberg equilibrium (HWE). Specifically, the H_obs_ was utilized to measure the genetic polymorphic degree of a specific loci, which is described as the observed proportion of heterozygous genotypes detected in a population (Sheriff and Alemayehu, 2018). The H_exp_, a fundamental statistical parameter used to estimate genetic diversity within populations, is referred to as the expected proportion of heterozygotes under HWE (Nei, 1973). The PIC has been reported as a statistical indicator for measuring the polymorphisms of genetic markers in a population (Botstein et al., 1980; Shete et al., 2000), which is actually determined using heterozygosity and number of alleles (Al-jumaah et al., 2012). The PM is defined as the probability of a match between two unrelated individuals selected at random (Fisher, 1951; Malaspinas et al., 2011). Closely related to PM, the PD is the probability of distinguishing two unrelated individuals (Tillmar, 2010). The PE can be treated as the probability of excluding a man falsely indicated as the biological father, which is a measure of efficiency in paternity testing (Cifuentes et al., 2006; Tillmar, 2010; Vandeputte, 2012). For X-STRs and Y-STRs, the gene diversity (GD), PM, haplotype diversity (HD), and haplotype discrimination capacity (DC) were calculated based on the methods of previous reports (Nei, 1987; Liu et al., 2020). In terms of the IISNPs, the STRAF software v1.0.5 was again utilized to estimate the forensic parameters, including H_obs_, H_exp_, PIC, PD, PE, PM, TPI, and HWE.

### Population Genetic Analyses

A total of four group sets of previously published population data were exploited as references to perform population genetic analyses and further dissect the ancestral components of the Northwest Hui group. The employed data are as follows: raw data of 26 populations for 93 IISNPs enlisted from the 1000 Genomes Project (https://www.internationalgenome.org/data), raw data of 17 Y-STRs for 58 populations collected from previous studies or the Y Chromosome Haplotype Reference Database (YHRD) database (https://yhrd.org/), raw data of 26 populations for 55 AISNPs gathered from the 1000 Genomes Project, and allele frequencies of 109 populations for 55 AISNPs assembled from previously published studies (Kidd et al., 2014; Pakstis et al., 2015; Pakstis et al., 2017; Pakstis et al., 2019a). The detailed reference population information and citations are listed in Supplementary Table S1.

Initially, population genetic analyses of the Northwest Hui group and the 26 reference populations (Auton et al., 2015) were performed based on the 93 overlapping IISNPs. A heatmap of allele frequencies for these 93 IISNPs in the Northwest Hui and 26 reference populations was plotted using the *R* software v3.3 (Team, 2016), which was hierarchically clustered based on the Euclidean distances. Principal component analysis (PCA) of these populations was conducted using the XLSTAT program (https://www.xlstat.com/en/) based on the allele frequencies of these 93 IISNPs. We also conducted a PCA plot of these populations at the individual level using the PLINK software v1.9 based on the raw data of these 93 IISNPs (Chang et al., 2015). The pairwise *D*_*A*_ genetic distances of these populations were calculated using the DISPAN program (http://www.personal.psu.edu/nxm2/dispan2.htm), and a rooted neighbor-joining (NJ) tree was further constructed using the MEGA software v6.0 (Tamura et al., 2013) based on the *D*_*A*_ genetic distances. Moreover, the population-specific divergence values of each loci in different intercontinental population sets were estimated using the informativeness for assignment (*I*_*n*_) statistic based on the allele frequencies of 93 IISNPs (Phillips, 2015). The *I*_n_ statistical analysis was originally introduced by Rosenberg et al. (2003), with the purpose of determining the amount of ancestry information provided by biallelic or multiallelic markers, which could be further utilized to evaluate the effectiveness of genetic markers in differentiating various populations. Next, the population genetic relationships between the Hui group and 58 worldwide populations were revealed from a paternal perspective based on 17 overlapping Y-STRs. The pairwise *R*_*st*_ values based on Y-STR haplotypes were generated using an online AMOVA tool (https://yhrd.org/amova). *R*_*st*_ is developed based on a stepwise mutation model to assess the genetic differentiations among populations (Balloux and Lugon-Moulin, 2002). Both heatmaps and histograms of the *R*_*st*_ values were performed using *R* software v3.3 (Team, 2016). Multidimensional scaling (MDS) was yielded based on R_st_ values using the Statistical Package for the Social Sciences (SPSS) 16.0 software. Eventually, the ancestral structure of the Northwest Hui group based on 55 AISNPs was explored using two sets of population data as the references. The PCA plot on individual level and STRUCTURE analysis were performed using the XLSTAT program (https://www.xlstat.com/en/) and ADMIXTURE software v1.3 (Alexander et al., 2009), respectively, based on the 55 AISNPs genotypes of the Northwest Hui group and the 26 reference populations. The ancestral component analysis of the Northwest Hui group was performed using ADMIXTURE software v1.3 (Alexander et al., 2009) on the basis of 55 AISNPs genotypes. The PCA, pairwise *D*_*A*_ genetic distances, and the rooted NJ tree of the Northwest Hui and other 109 reference populations were conducted using SPSS 16.0 software, the DISPAN program, and MEGA software v6.0 (Tamura et al., 2013), respectively, using the allele frequencies of 55 overlapping AISNPs.

## Results

### MPS Results for Three Genres of STRs and Three Kinds of SNPs

The MPS genetic data of the 90 male Hui individuals were genotyped using the primer mix B reagent of the ForenSeq™ DNA Signature Prep Kit. The genotyping results for three genres of STRs (27 A-STRs, 24 Y-STRs, and 7 X-STRs) based on both length- and sequence-based polymorphisms are presented in Supplementary Table S2. The total success rates of 27 A-STRs, 24 X-STRs, and 7 Y-STRs were 99.96%, 99.84%, and 99.81%, respectively. A total of 54 out of 58 STR loci were efficiently genotyped with a success rate of 100%, while the success rates of the remaining PentaE, DYS392, DYS448, and DXS7132 loci were 98.89, 98.89, 96.67, and 98.89%, respectively. The genotyping results for 94 IISNPs, 56 AISNPs, and 22 PISNPs are shown in Supplementary Table S3 with the success rates of 100%. As presented in Supplementary Table S4, for 230 genetic markers without an amelogenin locus, the total sequencing depth of 90 samples was 2,530,188; the average sequencing depths for each sample and each locus were 28,113.20 and 11,000.82, respectively; further, the average sequencing depth per locus for each sample was 122.23. In detail, as shown in Supplementary Figure S1, the sequencing depths of the A-STRs fluctuated from 209.97 (SD ± 166.79) at PentaE locus to 12,535.64 (±3,565.09) at TH01 locus. The minimum sequencing depth of the X-STRs was 91.13 (±30.97) at DXS10103 locus, whereas the maximum sequencing depth, observed at DXS10074 locus, was 2,540.82 (±827.35). For the Y-STRs, the DYS438 locus provided the highest sequencing depth as 10,176.43 (±3,039.40), while the DYS460 locus offered the lowest sequencing depth as 221.01 (±106.94). In terms of the 94 IISNP loci, the sequencing depths ranged from 77.14 (±21.23) at rs1736442 locus to 5,700.47 (±2,072.63) at rs8037429 locus. The maximum and minimum sequencing depths of 56 AISNPs were detected as 6,432.28 (±1,907.24) at rs7997709 locus and 208.23 (±57.93) at rs310644 locus, respectively. The sequencing depths of 22 PISNP loci were in the range from 430.57 (±120.85) at rs12821256 locus to 2,456.13 (±628.14) at rs2402130 locus.

### Forensic Parameters for Various Genres of Genetic Markers

**FIGURE 1:**
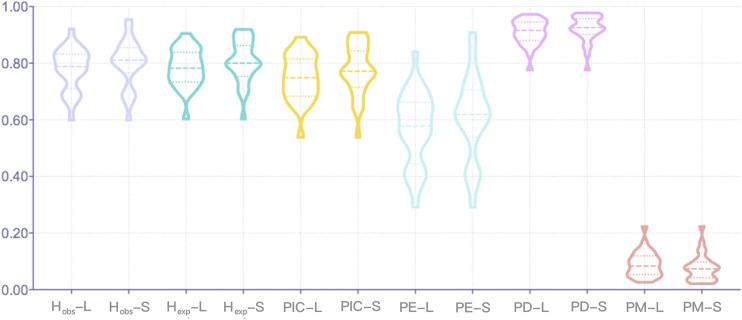
The forensic parameters of 27 A-STR loci at the length (L)- and sequence (S)-based levels. A-STR, autosomal short tandem repeat.

For the Northwest Hui group, the allelic polymorphisms and forensic parameters of 27 A-STR loci calculated on the basis of sequence- and length-based polymorphisms are presented in Supplementary Table S5. The number of length-based alleles fluctuated from 5 at D3S1358 and D4S2408 loci to 15 at D18S51, FGA, and Penta E loci, whereas the number of sequence-based alleles varied from 6 at D22S1045, TH01, and TPOX loci to 33 at D12S391 locus. HWE tests were conducted separately on sequence- and length-based A-STR loci. Before Bonferroni correction, it was observed that the TH01 locus deviated from the HWE for both sequence- and length-based polymorphisms; the vWA locus departed from the HWE for length-based polymorphisms; and the remaining 25 A-STR loci were in accordance with the HWE. The other forensic parameters are graphically presented in Figure 1. The H_obs_ values for length- and sequence-based STRs ranged from 0.6000 to 0.9222 and from 0.6000 to 0.9556, respectively, of which the lowest values were all observed at TPOX locus and the highest at D12S391 locus. The H_exp_ values for the length-based STRs spanned from 0.604 at TPOX locus to 0.906 at Penta E locus, and for sequence-based STRs varied from 0.604 at TPOX locus to 0.9200 at D12S391 locus. The average H_obs_ and H_exp_ values for the length-based polymorphisms were 0.7798 (±0.0744) and 0.7845 (±0.0670), respectively. For the sequence polymorphism level, the average H_obs_ and H_exp_ values were 0.7999 (±0.0829) and 0.8073 (±0.0747), respectively. The lowest PIC values were detected at TPOX locus with the value of 0.5387 for both length- and sequence-based polymorphisms, whereas the highest values of length- and sequence-based polymorphisms were 0.8928 at PentaE locus and 0.9095 at D12S391 locus, respectively. The TPOX locus revealed the lowest PE value for both length- and sequence-based STRs with the value of 0.2909, and the D12S391 locus exhibited the highest PE values for both length- and sequence-based STRs with values of 0.8410 and 0.9096, respectively. The combined PE values for the length- and sequence-based genotypes were 1–3.4332 × 10^–11^ and 1–1.0266 × 10^–12^, respectively. The PD values of all A-STR loci were larger than 0.7760 at TPOX locus for both length- and sequence-based polymorphisms, while all the PD values were less than 0.9741 at PentaE locus based on length polymorphisms and less than 0.9788 at D21S11 locus based on sequence polymorphisms. Additionally, the combined PD value observed at the length and sequence levels were 1–3.0634 × 10^–30^ and 1–8.0118 × 10^–33^, respectively. The PM values were in the range from 0.0259 (PentaE) to 0.2240 (TPOX) for length-based STRs and from 0.0212 (D21S11) to 0.2240 (TPOX) for sequence-based STRs. The TPOX locus showed the lowest TPI value as 1.2500 for both length- and sequence-based STRs, and the D12S391 locus presented the highest TPI values as 6.4286 for length-based STRs and 11.25 for sequence-based STRs.

Further, the haplotypic results and forensic parameters of 24 Y-STRs revealed from the perspective of length and sequence levels are presented in Supplementary Table S6. The GD values for length-based polymorphisms spanned from 0.4793 at DYS391 to 0.9718 at DYS385a-b loci, and for sequence-based polymorphisms from 0.4793 at DYS391 to 0.9903 at DYF387S1 locus. Eleven of the 24 Y-STRs exhibited discrepancies in GD values between length- and sequence-based Y-STRs, of which the GD values increased from 0.6324 to 0.6425 at DYS389I, 0.7813 to 0.9311 at DYS389II, 0.7610 to 0.7893 at DYS390, 0.5181 to 0.6774 at DYS437, 0.5912 to 0.6372 at DYS438, 0.7279 to 0.7391 at DYS439, 0.7498 to 0.8656 at DYS448, 0.8209 to 0.8272 at DYS570, 0.8599 to 0.8754 at DYS612, 0.8237 to 0.8569 at DYS635, and 0.6857 to 0.6932 at Y-GATA-H4 locus. No discrepancies were observed between the length- and sequence-based results for the DC, PM, and HD values, which were 1.0000, 0.0111, and 1.000, respectively. The length- and sequence-based haplotypes and forensic parameters for seven X-STRs in males are displayed in Supplementary Table S7. The GD values fluctuated from 0.5316 at DXS7423 to 0.8986 at DXS10135 locus for length-based polymorphisms and from 0.5316 at DXS7423 to 0.9176 at DXS10135 locus for sequence-based polymorphisms. For seven X-STR loci, different GD values for length- and sequence-based STRs were detected at four loci, including DXS8378, DXS7132, DXS10103, and DXS10135. The length-based polymorphisms were disclosed the same DC, PM, and HD values as the sequence-based polymorphisms, which were 1.0000, 0.0111, and 1.0000, respectively.

The allele frequencies and forensic parameters of 94 IISNPs are presented in Supplementary Table S8. The minor allele frequencies (MAF) approximately ranged from 0.1056 (rs2040411, T; rs733164, G) to 0.4944 (rs1028528, A). The MAF emerged 32.98% of the total loci for A alleles, 22.34% for C alleles, 20.21% for G alleles, and 24.47% for T alleles. The H_obs_ and H_exp_ values varied from 0.1889 (rs1355366, rs2056277, rs2107612, and rs733164) to 0.5778 (rs722290) and from 0.1900 (rs2056277 and rs740910) to 0.5030 (rs3780962, rs891700, rs2269355, and rs907100) with the average values of 0.4249 (±0.0958) and 0.4330 (±0.0842), respectively. The PIC values varied from 0.1710 at rs2056277 and rs740910 loci to 0.3750 at rs891700 locus with an average value of 0.3343 (±0.0532). The PD values spanned from 0.3242 (rs2056277) to 0.6649 (rs214955) with average value of 0.5708 (±0.0795), and the combined PD value for the 94 IISNPs was 1–7.3652 × 10^–36^. The PE values ranged from 0.0268 (rs1355366, rs2056277, rs2107612, and rs733164) to 0.2651 (rs722290) with average value of 0.1403 (±0.0579). The lowest PM value was 0.3351 at rs214955 locus, whereas the highest value was 0.6758 at rs2056277 locus; and the average value was 0.4292 (±0.0795). The TPI values were in the range from 0.6164 (rs1355366, rs2056277, rs2107612, and rs733164) to 1.1842 (rs722290), and the average value was 0.8916 (±0.1354).

### STR Sequence Variations Observed Using the MPS Method

The sequence- and length-based alleles and corresponding frequencies of 58 STRs (27 A-STRs, 24 Y-STRs, and 7 X-STRs) for the Northwest Hui group are listed in Supplementary Table S9. Additional alleles with sequence variants were revealed by using the MPS platform, which were indistinguishable using the CE method. In contrast to the length-based alleles, increasing rates of detected alleles on the sequence level were observed, which are presented in Supplementary Table S10. On the basis of length polymorphism alone, 451 distinct alleles were identified from 58 STRs in 90 unrelated Hui individuals. When the sequence variants were taken into consideration, the allelic diversities of 31 STRs, including 15 A-STRs, 12 Y-STRs, and 4 X-STRs, increased obviously, leading to a total allele number of 652 for 58 STRs in the Northwest Hui group. In detail, the allele numbers for A-STRs in the Hui group ranged from 5 (D3S1358 locus) to 15 (D18S51, FGA and PentaE loci) on the length polymorphism level, whereas 6 (D22S1045, TH01 and TPOX loci) to 33 (D12S391 locus) alleles were observed on the sequence level, which contributed 1 to 23 additional alleles. The D12S391 locus exhibited the most diversity with 230% increase in ratio. The allele numbers in four STRs, including D2S1338 (170.00%), D21S11 (158.33%), D13S317 (125.00%), and D3S1358 (120.00%) loci, increased by more than double. For the Y-STRs loci, a total of 155 and 231 different alleles were identified on the basis of length and sequence polymorphisms, respectively. The highest allelic numbers were 12 at DYS385a-b loci for the length-based level and 33 at DYF387S1 locus for the sequence-based level. The increased ratios of allelic numbers in three Y-STRs exceeded 100%, including DYF387S1 (266.67%), DYS389II (242.86%), and DYS448 (150.00%) loci. Nine Y-STRs displayed 16.67–83.33% extra sequence-based alleles compared with length-based alleles. Four X-STRs, DXS10135, DXS10103, DXS8378, and DXS7132 loci, showed both length and sequence polymorphisms with the increasing ratios ranging from 14.29% to 57.89%. No additional alleles were identified based on sequence polymorphisms in 12 A-STRs, 12 Y-STRs, and 3 X-STRs compared with the length-based polymorphisms, thus providing the same recognition capability as the traditional CE approach.

### Phenotypic Predictive Analysis

The phenotypic SNPs utilized in this study mainly focus on pigmentation, including hair and eye colors. The predicted phenotype results for male individuals from the Northwest Hui group are displayed in Supplementary Table S11 and Supplementary Figure S2. The hair colors contained four possible traits, including brown, red, black and blond colors, and the eye colors encompassed three possible traits, including brown, blue and intermediate colors. The tested 90 male individuals from the Northwest Hui group were predominantly revealed with black hair and brown eyes, and the percentages of black hair and brown eyes for all individuals were in the range from 52% to 96%, and 87% to 100%, respectively. The predicted results are roughly in accordance with the phenotypes of the studied Hui group.

### Genetic Relationships Between the Northwest Hui Group and Reference Populations

#### Genetic Differentiation Analyses Between the Northwest Hui Group and Reference Populations Based on IISNPs

The genetic differentiations between the Northwest Hui group and worldwide reference populations were analyzed based on the overlapping IISNPs loci. The reference data were obtained from 2,504 individuals of 26 populations in the 1000 Genomes Project. In contrast with the 94 IISNPs provided by the ForenSeq™ DNA Signature Prep Kit, one locus named rs938283 was not found in the 1000 Genomes Project, thus leading to the use of 93 overlapping IISNPs in the following analyses. Serial plots, including a heatmap of ancestral allele frequencies, a NJ tree, two PCA plots, and a plot of population-specific divergences based on *I*_n_ statistic, were constructed, with the intention of determining the genetic discrimination abilities of these 93 IISNPs among the Northwest Hui group and reference populations, as well as the individual identification performances of these IISNPs in the Northwest Hui group.

**FIGURE 2:**
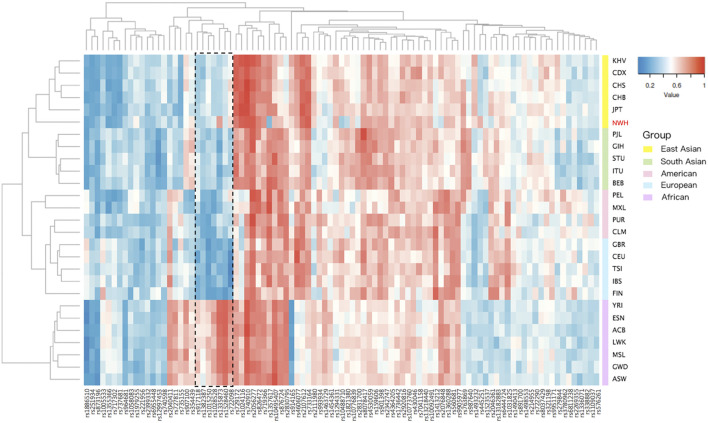
Cluster heatmap constructed based on allele frequencies of 93 overlapping IISNP loci in the Northwest Hui and 26 reference populations. IISNP, identity-informative single nucleotide polymorphism.

The allele frequencies of 93 IISNPs were utilized to generate a cluster heatmap, facilitating the intuitive visualization of polymorphic distributions for these 93 same IISNPs in the Northwest Hui group and reference populations. As presented in Figure 2, the deeper blue indicated lower allelic frequency values, whereas the deeper red represented higher-frequency values. Populations were divided by different continental origins, including African, American, East Asian, European, and South Asian clusters, whereas some clusters exhibited different patterns of allelic frequency distributions. In particular, the frequency distributions in African populations were evidently distinct from those in the other four intercontinental clusters. For example, one set of IISNP loci marked using the dotted box in Figure 2, including the rs917118, rs1382387, rs1015250, rs1028528, rs1335873, rs1528460, and rs722098 loci, overwhelmingly displayed red in African populations, while they presented blue in the remaining populations from the other four continents. Additionally, the allelic frequency distribution in the Northwest Hui group showed the most similar pattern to East Asian populations, in accordance with the dendrogram classifications based on Euclidean distances in Figure 2. The MAF values of the Northwest Hui group and reference populations are plotted based on the allelic frequencies of these IISNPs in Supplementary Figure S3.

**FIGURE 3:**
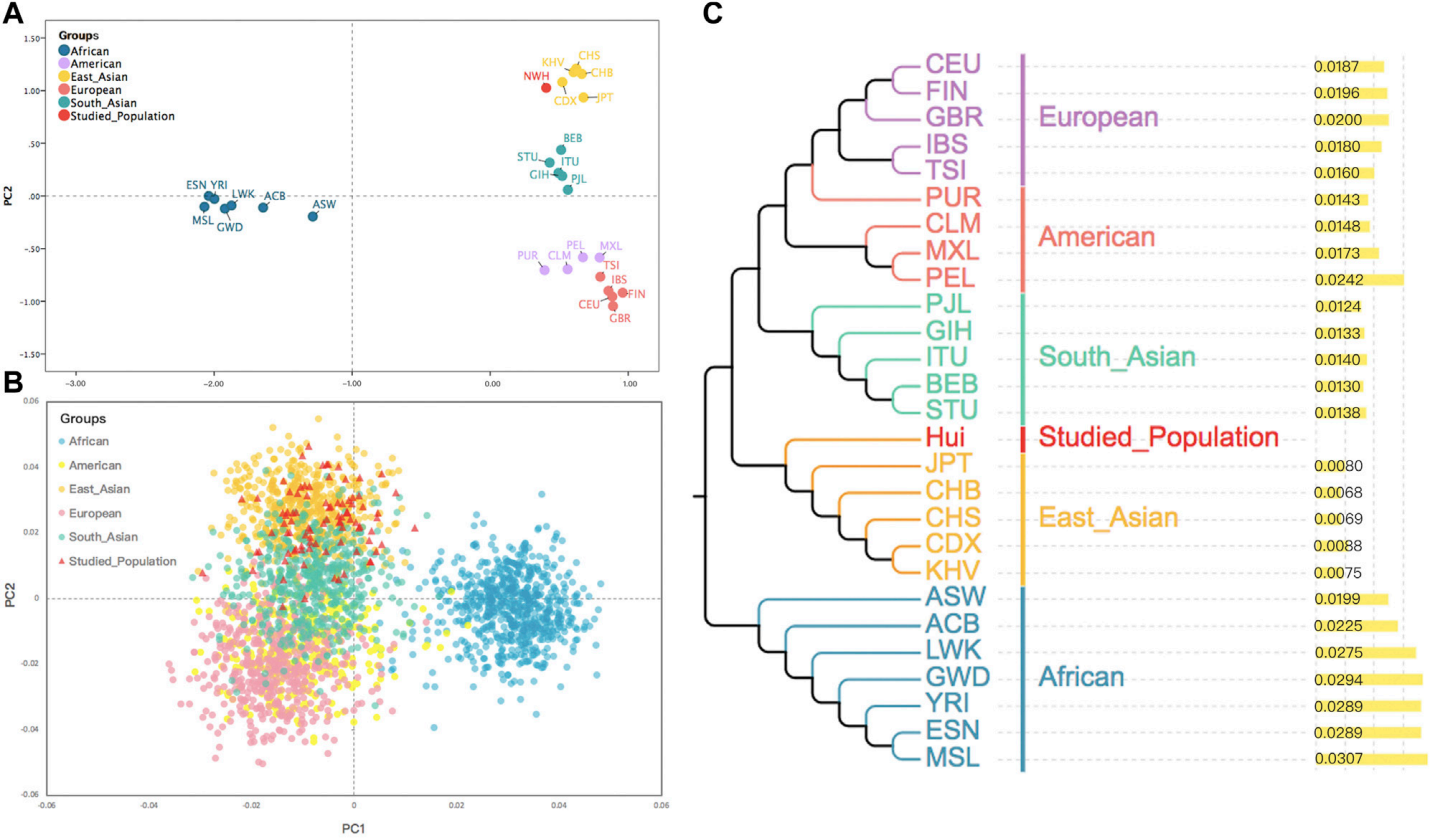
**(A)** A PCA plot of the Northwest Hui group and the 26 reference populations based on the allele frequencies of these 93 same IISNPs. **(B)** A PCA plot for individuals from the Northwest Hui group and the 26 reference populations calculated based on the raw data of these 93 same IISNPs. **(C)** A neighbor-joining tree constructed based on the *D*_*A*_ genetic distances of the Northwest Hui group and the 26 reference populations. PCA, principal component analysis; IISNPs, identity-informative single-nucleotide polymorphisms.

To further inspect whether the IISNP genetic markers could reveal the genetic relationships between the Northwest Hui group and worldwide reference populations, two PCA plots and one NJ tree were constructed. As presented in Figure 3A, the genetic relationships among populations were revealed by the first and second principal components accounted for 48.58% and 20.22%, respectively. According to the first discrimination component, all the populations were segregated into two group sets, i.e., the African group (deep blue) and the non-African group. From the perspective of the second discrimination component, the non-African populations were further categorized into four subgroups. One subgroup was distributed in the top right corner of the first quadrant, including populations from East Asia (yellow) and the Northwest Hui group (red); South Asian populations (green) belonging to another subgroup were located directly beneath the East Asian populations in the bottom right corner of the first quadrant; European populations (pink) were clustered as a subgroup in the lower right corner of the fourth quadrant; and American populations (purple) were scattered between the South Asian and European subgroups in the fourth quadrant. In addition, to dissect the genetic relationships more deeply, a PCA plot on the individual level was constructed. As shown in Figure 3B, the dots in various colors represented the individuals deriving from different biogeographical regions. All individuals were divided into two large cluster groups on the first principal component. Africans in deep blue occupied the right-hand side of the plot as the first cluster. The other non-African cluster was further dispersed into three sub-clusters on the second principal component, namely, East Asians in orange, South Asians in green, and Europeans in pink, which superimposed without a clear boundary. The Americans in yellow were mainly clustered with South Asians and Europeans. Individuals from the Northwest Hui group (red) predominantly overlapped with East Asians, while sporadic Hui individuals were scattered among the South Asians. A NJ tree coupled with a histogram was subsequently constructed based on the *D*_*A*_ values in Figure 3C. All populations were divided into four distinct sub-branches, which were the European and American populations for the first, South Asian populations for the second, the East Asian populations for the third, and the African populations for the fourth sub-branch. The cluster distributions of all populations were roughly concordant with their geographical regions, except for four American populations. Further, the Northwest Hui group was evidently clustered with East Asian populations and displayed the lowest divergencies with Han populations, including the CHB (*D*_*A*_ = 0.0068) and CHS (*D*_*A*_ = 0.0069) populations. Obviously, populations from Africa revealed the largest genetic distances from the Northwest Hui group with the *D*_*A*_ values ranging from 0.0199 to 0.0307.

**FIGURE 4:**
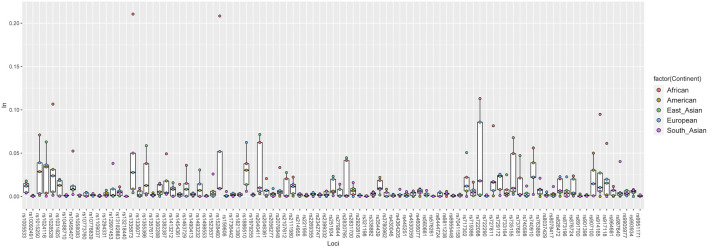
A plot of population-specific divergences for five intercontinental population groups performed based on *I*_n_ statistic.

Eventually, to determine the ability of the 93 IISNP loci on distinguishing populations, a plot of population-specific divergences for the five intercontinental clusters was constructed based on the *I*_n_ statistic. In Figure 4, populations from one continent had one corresponding *I*_n_ value at each IISNP locus, thus, there were five intercontinental *I*_n_ values at each IISNP locus. African populations generated the largest *I*_n_ values at 30 IISNP loci, ranging from 0.0200 at rs901398 locus to 0.2105 at rs1335873 locus, in comparison with the remaining intercontinental populations. African populations with relatively high *I*_n_ values always tended to separate from American, East Asian, European, and South Asian populations, especially at four loci (rs1335873, rs1528460, rs722098, and rs1028528) revealing the highest discrepancy *I*_n_ values (*I*_n_ > 0.1). Additionally, American populations encompassed 13 loci showing the largest discrepancy values, and the *I*_n_ values of American populations at these 13 loci varied from 0.0004 at rs13218440 locus to 0.0219 at rs354439 locus. A total of 19 loci showed the largest discrepancy values in East Asian populations, ranging from 0.0005 at rs576261 locus to 0.0715 at rs2040411 locus. European and South Asian populations exhibited the largest discrepancy values at 11 and 20 loci, respectively, of which the *I*_n_ values were in the range from 0.0015 (rs6955448) to 0.0623 (rs1886510) and from 0.0018 (rs722290) to 0.0403 (rs987640), respectively. The *I*_n_ values in American populations were in the range from 0.0000 at six loci (rs873196, rs914165, rs2399332, rs10092491, rs763869, and rs2056277) to 0.0389 at rs740910 locus with an average value of 0.0052 (±0.0084). East Asian populations generated *I*_n_ values ranging from 0.0000 at seven loci (rs8078417, rs2046361, rs13218440, rs1058083, rs1336071, rs1109037, and rs9951171) to 0.0715 at rs2040411 locus with an average value of 0.0091 (±0.0157). Nine loci, including rs2831700, rs2107612, rs445251, rs2342747, rs13218440, rs321198, rs993934, rs3780962, and rs2830795, presented the lowest discrepancy values (*I*_n_ = 0.0000) in European populations, while rs722098 locus yielded the highest discrepancy value (*I*_n_ = 0.0858), and the average *I*_n_ value was 0.0245 (±0.0190). The *I*_n_ values in South Asian populations varied from 0.0000 at eight loci (rs6444724, rs873196, rs917118, rs1413212, rs2830795, rs2269355, rs576261, and rs717302) to 0.0500 at rs1335873 locus with an average value of 0.0140 (±0.0118). The African populations offered the lowest discrepancy value (*I*_n_ = 0.0000) at eight loci (rs10488710, rs891700, rs338882, rs1493232, rs576261, rs445251, rs159606, and rs13218440), whereas the highest value was observed at rs1335873 locus (*I*_n_ = 0.2105), and the average value was 0.0198 (±0.0373).

#### The Comparisons of Y-STR Haplotype Polymorphisms Between the Northwest Hui Group and Reference Populations

The patrilineal genetic data of 59 populations, the Northwest Hui group and 58 reference populations, were employed in this study, which could be classified into six different biogeographical group sets, including 44 populations from East Asia, seven populations from West Asia, three populations from Central Asia, two populations from Africa, two populations from Europe, and one population from Australia. A total of 17 shared Y-STR loci were employed from these 59 populations, including DYS19, DYS389I, DYS389II, DYS390, DYS391, DYS392, DYS393, DYS385a, DYS385b, DYS437, DYS438, DYS439, DYS448, DYS456, DYS458, DYS635, and YGATAH4. It is worth mentioning that three loci (DYS393, DYS456, and DYS458) from the abovementioned 17 Y-STR loci were absent in the 24 Y-STR markers from the ForenSeq™ DNA Signature Prep Kit. The genotype profiles of these three loci in the Northwest Hui group were replenished using the PCR and CE-based method.

**FIGURE 5:**
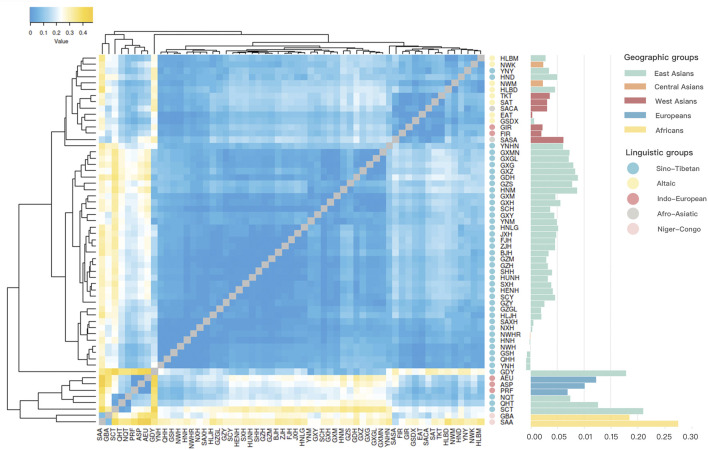
A cluster heatmap constructed based on the pairwise *R*_*st*_ values of the Northwest Hui group and 58 reference populations, in combination with a histogram of pairwise *R*_*st*_ values between the Northwest Hui group and other reference populations in the right side.

As shown in Figure 5, a heatmap was constructed using the pairwise *R*_*st*_ values calculated based on the raw Y-STR data from the worldwide populations. The gradually deepening yellow indicated the increasing *R*_*st*_ values, whereas the deepening blue indicated the decreasing *R*_*st*_ values. In the heatmap, populations were labeled according to different linguistic families, containing eight populations from the Altaic family in yellow, two populations from the Afro-Asiatic family in grey, five populations from the Indo-European family in red, 42 populations from the Sino-Tibetan family in blue, and two populations from the Niger-Congo family in pink. All the Hui groups from Chinese different regions, including the studied Northwest Hui group (NWH), Ningxia Hui (NXH), Shaanxi Hui (SAXH), Henan Hui (HNH), Gansu Hui (GSH), Qinghai Hui (QHH) and Yunnan Hui (YNH), except for Sichuan Hui (SCH) were primarily clustered together, then gathered with the populations from East Asia, and subsequently grouped with Central and West Asian populations in deeper blue color.

To display the genetic relationships more clearly, a histogram of pairwise *R*_*st*_ values between the Northwest Hui group and the reference populations was plotted on the right-hand side of the heatmap. The Northwest Hui group retained extremely low genetic distances (*R*_*st*_ < 0.01) with other reference Hui groups from Chinese different regions, with the exception of SCH. In detail, four reference Hui groups from Chinese different regions, including GSH (*R*_*st*_ = −0.0068), HNH (*R*_*st*_ = −0.0022), QHH (*R*_*st*_ = −0.0082), and YNH (*R*_*st*_ = −0.0081), displayed negative *R*_*st*_ values; two reference Hui groups, NXH (*R*_*st*_ = 0.0041) and SAXH (*R*_*st*_ = 0.0062), showed extremely low genetic distances; and one reference Hui group, SCH (*R*_*st*_ = 0.0371), exhibited the lower genetic differentiation (0.01 < *R*_*st*_ < 0.05) from the Northwest Hui group. Intriguingly, the studied Hui group revealed clearly lower genetic differentiations from the Central and West Asian populations, especially for East Anatolian Turkey (EAT) (*R*_*st*_ = 0.0038). It is worth mentioning that one Chinese group named Gansu Dongxiang (GSDX) (*R*_*st*_ = 0.0072) also exhibited extremely low genetic distance with the Northwest Hui group.

**FIGURE 6:**
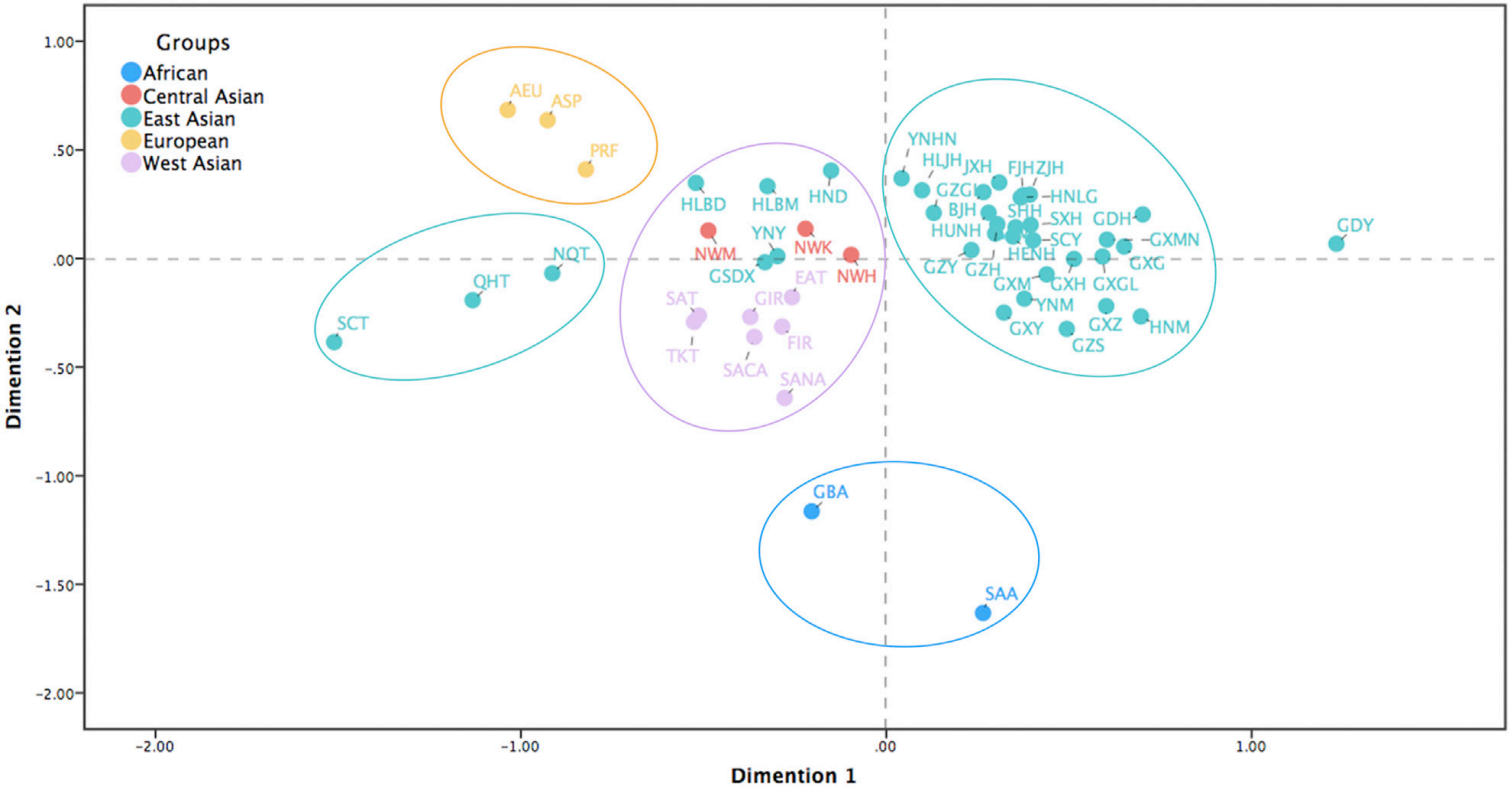
MDS analysis for the Northwest Hui group and the reference populations without the reference Hui groups from Chinese different regions based on pairwise *R*_*st*_ values. MDS, multidimensional scaling.

To further verify the genetic relationships between the Northwest Hui group and the reference populations, we conducted a MDS analysis for all the populations without the reference Hui groups based on pairwise *R*_*st*_ values. In Figure 6, the populations were roughly divided into several clusters as follows. Two African populations (blue) were scattered in the bottom of the plot, three European populations (yellow) were in the upper left corner, and the majority of the East Asian populations (green) were gathered in the upper right side. Evidently, the Northwest Hui group was located between East and West Asian populations, but was more prone to blend with West Asian (purple), Central Asian (red), and several East Asian (green) populations, including the Arab (SACA and SASA), Turkish (EAT, TKT, and SAT), and Iranian (FIR and GIR) populations from West Asia; the Northwest Kazakh (NWK) and Northwest Mongolian (NWM) groups from Central Asia; and Hulunbuir Mongolian (HLBM), Hulunbuir Daur (HLBD), GSDX, and other populations from East Asia.

#### Population Genetic Analyses Based on AISNPs

In the present study, we employed two sets of population data on the basis of the 55 overlapping AISNPs to uncover biogeographical ancestral information for the Northwest Hui group, including 26 reference populations from the 1000 Genomes Project and 109 reference populations from previous studies.

**FIGURE 7:**
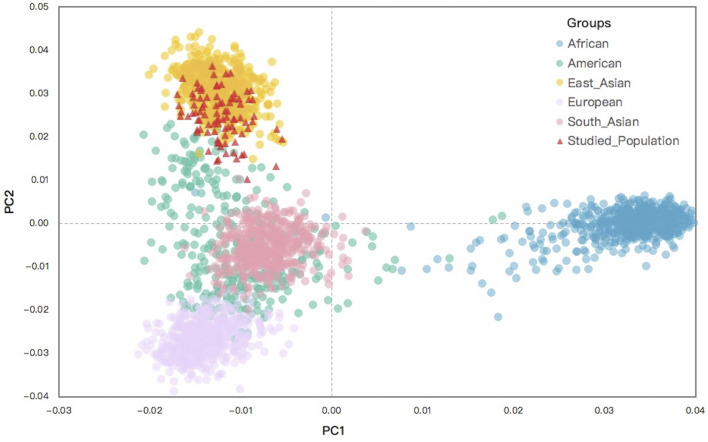
A PCA plot for individuals from the Northwest Hui group and the 26 reference populations based on the raw data for the same 55 AISNPs. PCA, principal component analysis; AISNPs, ancestry-informative single-nucleotide polymorphisms.

The PCA analysis of the Northwest Hui group and the 26 reference populations was constructed, from which the raw population data of 55 same AISNPs were available. To obtain a more intuitive display, the first two principal components were adopted to perform the PCA plot. As presented in Figure 7, the clustering pattern of individuals from five continents were revealed, preliminarily clarifying the ancestral determination performance of these 55 AISNPs. Africans (blue) located on the right-hand side of the plot were clearly distinguished from the other populations based on the first principal component. When the second principal component was taken into consideration, three clusters of individuals belonging to different continental regions were observed, which were East Asians (orange), South Asians (pink), and Europeans (purple). However, individuals from America (green) were largely overlapped with individuals from South Asia and Europe. The studied Hui individuals (red) were mainly superimposed with the East Asians.

**FIGURE 8:**
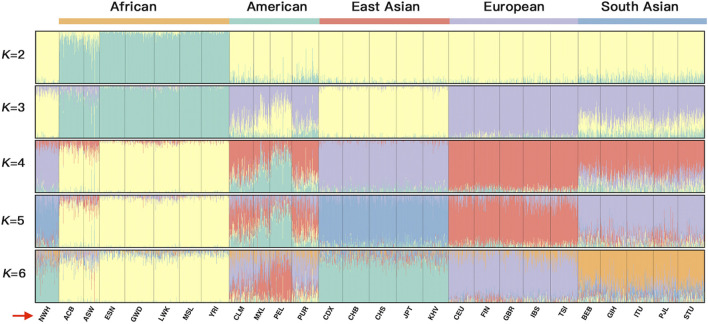
A STRUCTURE plot for the 26 reference populations and the Northwest Hui group based on the raw population data for 55 AISNPs from *K* = 2 to *K* = 6. The *K* values are equivalent to the predicted ancestral components shown in different colors. AISNPs, ancestry-informative single-nucleotide polymorphisms.

To explicitly demonstrate the ancestral admixture patterns, we executed a STRUCTURE algorithm from *K* = 2 to *K* = 6 for the 26 reference populations and the Northwest Hui group based on the raw population data of 55 AISNPs in Figure 8. The different *K* values were equivalent to the number of predicted ancestral components in different colors. Each individual was denoted by a vertical line partitioned into several segments corresponding to the contributions of different ancestral components. For example, when *K* = 2 (top line, Figure 8) was taken into consideration, all individuals were composed of two presumed ancestral components (yellow and green). Only populations from Africa, mainly covered by the green component, were distinctly separated from the non-African populations indicated by the large proportion of yellow. An additional ancestral component in purple was added at *K* = 3, which was the optimal number of assumed ancestral components. In this case, the population clusters dominated by green, yellow, and purple appertained to Africa, East Asia, and Europe, respectively. Populations from America and South Asia exhibited similar ancestral components, the mixture of purple, yellow, and green, yet the proportions of the ancestral components were discrepant between the American and South Asian populations. At *K* = 3, the Northwest Hui group was dominated by yellow component and less purple component, unveiling an analogous admixture pattern with East Asian populations. Further, at *K* = 4, three population clusters from Africa, Europe, and East Asia were mainly distinguished by yellow, red, and purple components, respectively; an extra ancestral component in green could readily distinguish American populations from South Asian populations.

**FIGURE 9:**
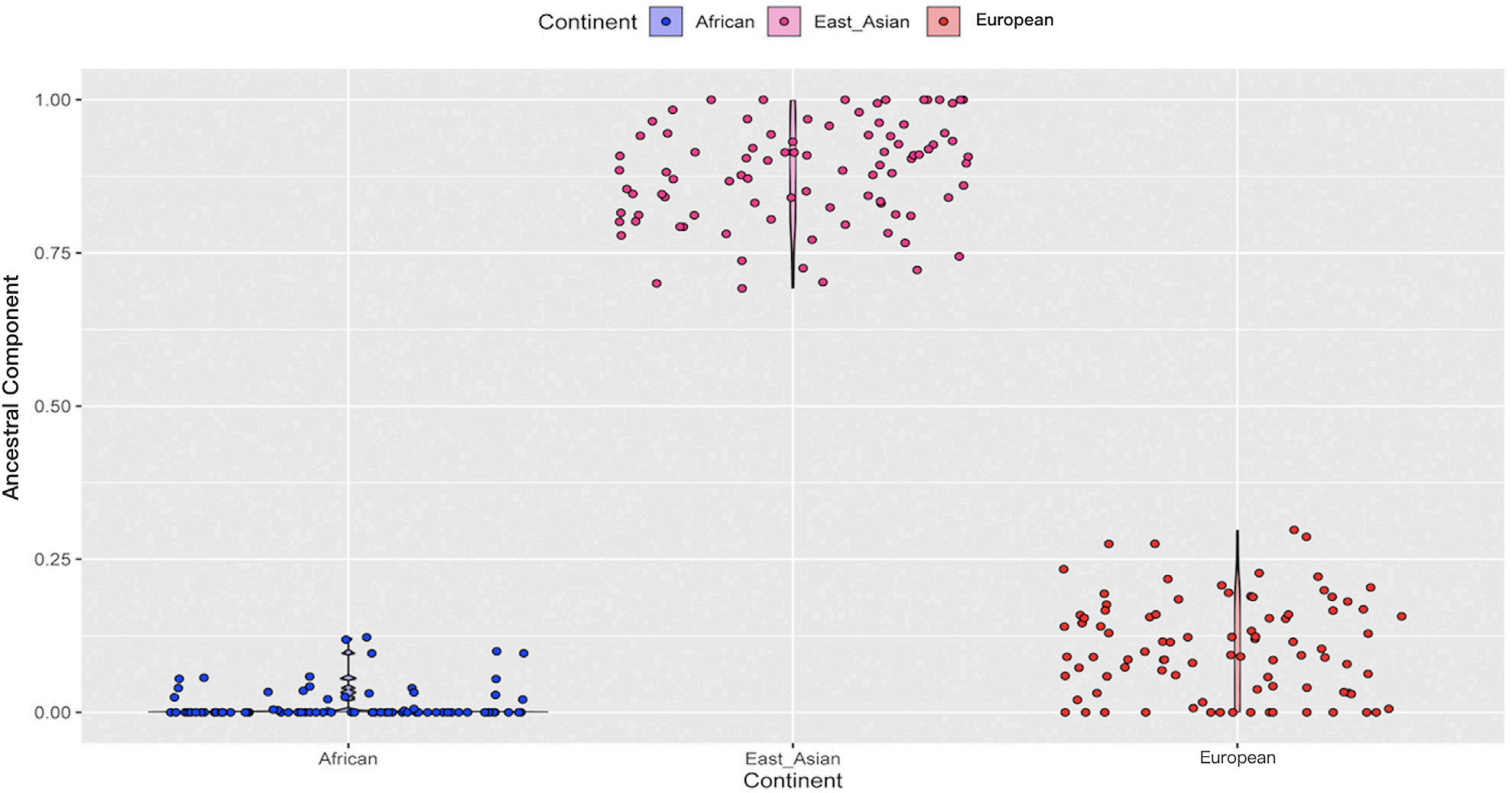
The prediction of ancestral components for the Northwest Hui group on the basis of 55 AISNPs genotypes. AISNPs, ancestry-informative single-nucleotide polymorphisms.

Based on the results of the STRUCTURE analyses, we subsequently conducted the prediction of ancestral components for the Northwest Hui group in Figure 9. A total of 90 Hui individuals represented by blue dots exhibited the lowest African ancestral component. On the contrary, 90 Hui individuals (pink dots) revealed the highest East Asian ancestral component, and the percentages of East Asian components exceeded 75% in the vast majority of Hui individuals. The Hui individuals shown with red dots disclosed small proportions of European ancestral components, with the percentages generally below 25%.

**FIGURE 10:**
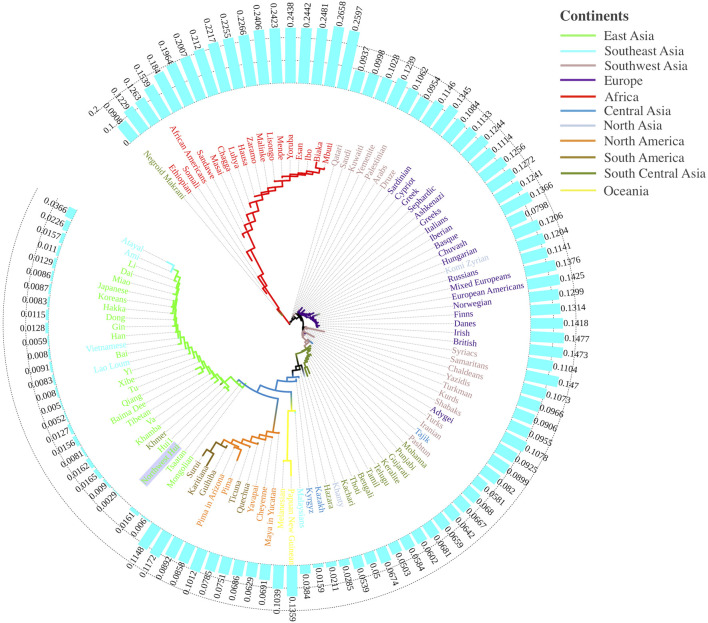
A neighbor-joining tree conducted based on Nei’s *D*_*A*_ values for the Northwest Hui group and 109 reference populations. A histogram of the *D*_*A*_ values between the Northwest Hui group and the reference populations distributed around the phylogenetic tree.

To comprehensively dissect the genetic structure of the Northwest Hui group, we subsequently implemented the data from 109 worldwide populations as the references, from which the population frequency data of 55 AISNPs were available. The Nei’s *D*_*A*_ values among the studied Hui group and the 109 reference populations were evaluated and utilized to construct a NJ tree. As shown in Figure 10, the NJ tree was encircled by the histogram of *D*_*A*_ values between the Northwest Hui group and the reference populations. For the NJ plot, populations from different continents tended to congregate corresponding to their biogeographical regions, mainly including populations from East Asia (green sub-branches), America (orange and brown sub-branches), South Asia (dark green, pink and light blue sub-branches), Europe (purple sub-branches), and Africa (red sub-branches). The Northwest Hui group was chiefly assembled with populations from East Asia. In detail, many Chinese populations exhibited extremely low genetic differentiations (*D*_*A*_ < 0.01) with the Northwest Hui group. The smallest genetic distance was observed between the studied Hui group and the reference Hui group with *D*_*A*_ = 0.0029, followed by the Xibe group with *D*_*A*_ = 0.0050, the Tu group with *D*_*A*_ = 0.0052, the Southern Han population with *D*_*A*_ = 0.0059, the Mongolian group with *D*_*A*_ = 0.0060, the Yi group with *D*_*A*_ = 0.0080, the Tibetan group with *D*_*A*_ = 0.0081, the Hakka group with *D*_*A*_ = 0.0083, and the Bai group with *D*_*A*_ = 0.0091. In addition, three Southeast Asian populations (Vietnamese, Lao Loum, and Khmer) and two East Asian populations (Japanese and Korean) also displayed extremely low genetic distances with the Northwest Hui group. Populations from North America, South America, North Asia, and Europe predominantly manifested moderate differentiations (0.05 < *D*_*A*_ < 0.15) from the Northwest Hui group, whereas most populations from Africa primarily differentiated from the Northwest Hui group with large genetic distances (0.15 < *D*_*A*_ < 0.25).

**FIGURE 11:**
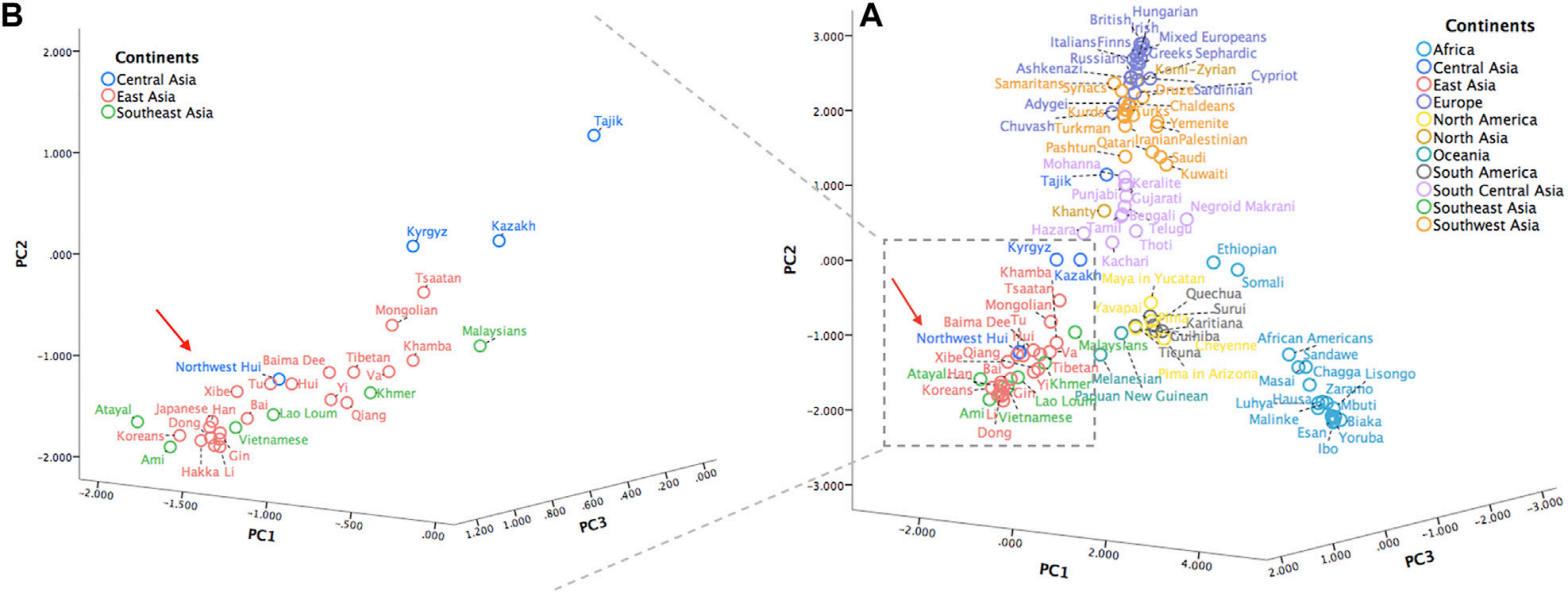
**(A)** A PCA plot of the Northwest Hui group and 109 reference populations carried out based on the frequency data of 55 AISNPs. **(B)** A PCA plot of the Northwest Hui group and populations from East, Southeast, and Central Asia. PCA, principal component analysis; AISNPs, ancestry-informative single-nucleotide polymorphisms.

To gain more insight into the population clustering pattern, a PCA plot of the Northwest Hui group and 109 reference populations was performed based on the frequency data of 55 identical AISNPs? (Figure 11A). The first principal component occupied 40.60% of the total variations, followed by the second principal component accounting for 37.97% and the third principal component accounting for 8.48%. Five population clusters, including populations from Africa (light blue), South Central Asia (light purple), Southwest Asia (orange), Europe (deep purple) America (yellow and grey), were clearly distinguished from each other. However, populations from East Asia (red), Southeast Asia (green), and Central Asia (blue) partially overlapped in the bottom left corner. To obtain clear insights of the relationships between the Northwest Hui group and the neighboring populations in the plot, another PCA plot was constructed (Figure 11B), concentrating on the populations from East, Southeast, and Central Asia. As anticipated, the studied Hui group was clustered with the majority of the East Asian populations and, more specifically, with the reference Hui and Tu groups.

## Discussion

### Sequencing Performance

Considering all 231 genetic markers, the sequencing depths for Northwest Hui individuals roughly fluctuated from 77.14 (±21.23) at rs1736442 IISNP locus to 12,535.64 (±3,565.09) at TH01 locus. A disequilibrium in sequencing depth was clearly observed among the different kinds of genetic markers. These sequencing results were within the acceptable criteria defined by the manufacturer and approximately consistent with previous MPS-related studies (Churchill et al., 2017; Guo et al., 2017; Köcher et al., 2018; Hollard et al., 2019; Hussing et al., 2019; Fan et al., 2020). Specifically, partial genetic markers presenting the lowest sequencing depths in this study, such as DYS460, DXS10103, and rs1736442, were also reported to perform poorly in other studies (Almalki et al., 2017; Guo et al., 2017; Jäger et al., 2017; Silvia et al., 2017; Xavier and Parson, 2017; Hollard et al., 2019; Fan et al., 2020). It was speculated that the constant low detection of these markers might be in relation to amplicon length, low (below recommendations) copy number input DNA, inaccurate library quantification, or overmultiplexing of samples (Guo et al., 2017).

### Forensic Parameter Efficiencies for Various Genres of Genetic Markers

No significant departure from the HWE expectation was observed following the Bonferroni correction of 27 A-STR loci. In terms of the other forensic parameters of 27 A-STRs shown in Figure 1, the sequence-based polymorphisms provided relatively higher median values in comparison with the length-based polymorphisms, except for the PM parameter, which indicated that the A-STR loci detected at the sequence level might contain more genetic information compared to those at the length level. Previous reports in the literature corroborate that genetic markers with heterozygosity values over 0.5 within populations are appropriate for genetic diversity studies (Dávila et al., 2009; Sheriff and Alemayehu, 2018), illustrating that these 27 A-STR loci with high H_obs_ and H_exp_ values at both length and sequence levels were suitably informative for genetic studies. Additionally, we also discovered that the average H_obs_ values were close to, although slightly lower than, the average H_exp_ values from either the length or sequence perspective, possibly indicating no overall loss in heterozygosity (Araújo et al., 2006). The PIC values at the length and sequence levels were both greater than 0.5; according to Marshall et al., a locus revealing a PIC >0.5 could be treated as highly informative and as a polymorphic marker for genetic characterization and diversity studies (Marshall et al., 1998). The average PM value generated using the sequence-based method was lower than that generated using the length-based method, indicating that sequence-based genotypes might contain more genetic polymorphisms than length-based genotypes. The combined PD value observed at the sequence level was higher than that at the length level, denoting that sequence-based STRs could provide more discrimination power for individual identifications. The combined PE value for the sequence-based genotypes was greater than that for the length-based genotypes, indicating that more genetic variations revealed by sequencing increased the exclusive ability for parentage identification.

When analyzing 94 IISNPs in the Northwest Hui group, only three loci, rs1360288, rs2107612, and rs214955, significantly deviated from the HWE expectations, which were further conformed to the HWE expectations after Bonferroni corrections. The average values of other forensic parameters, including H_obs_, H_exp_, PIC, PD, PE, and TPI, were overwhelmingly lower than those of A-STRs. However, the combined PD value for the 94 IISNPs was higher than those of the 27 A-STRs at both the length and sequence levels. Previous reports also claimed that these 94 IISNPs could be comparable with the 27 A-STRs considering the discrimination power for individual identification (Wendt et al., 2016; Li et al., 2018; Delest et al., 2020; Fan et al., 2020).

### STR Sequence Variations Observed Using the MPS Method

Compared with STR alleles characterized by the lengths of repeat regions, the detection of possible sequence variations in these repeat regions can effectively increase the polymorphism level of these alleles. In this study, the Northwest Hui group presented plentiful sequence variations on the basis of the MPS data. Specifically, the numbers of sequence-based alleles at eight STR loci were more than doubled in comparison with those of the length polymorphic alleles, including D3S1358, D13S317, D21S11, D2S1338, D12S391, DYS448, DYS389II, and DYF387S1, which were partially consistent with previously published studies (Gettings et al., 2015; Gettings et al., 2016; Novroski et al., 2016; Delest et al., 2020). For example, Novroski et al. claimed that D2S1338, D12S391, and D21S11 loci provided the most significant contribution to increasing allele diversity via sequence variations in repeat regions, focusing on African American, Caucasian, Hispanic, and Chinese populations (Novroski et al., 2016). D12S391 and DYF387S1 were determined to be the most polymorphic loci for the A-STRs and Y-STRs in French populations, respectively (Delest et al., 2020). These sequence variations enhanced the forensic efficiencies of these STR markers in the forensic applications, such as individual identification and kinship testing, which were generally consistent with previous studies (Hussing et al., 2019; Khubrani et al., 2019; Delest et al., 2020).

### Genetic Relationships Between the Northwest Hui Group and Reference Populations

#### The Performances of IISNPs in Population Genetics

As depicted in Figure 2, certain discrepancies the allelic frequency distributions among the intercontinental populations were observed, especially for partial IISNP loci marked by the dotted box. However, the SNP loci utilized for forensic individual identifications are reported to display little allele frequency differentiation and high heterozygosity among the applied populations (Kidd et al., 2006; Yousefi et al., 2018). Further, MAF can also be considered when screening the SNP loci for individual identifications, which is defined as the frequency of the least common allele for each genetic marker in a given population (Huang et al., 2018; Yousefi et al., 2018). Various MAF thresholds were implemented for the selection of optimal IISNPs in previously published research. For example, Yousefi et al. chose the SNP loci with MAF value of more than 0.2 as one of the criteria for the individual identification (Yousefi et al., 2018). Huang et al. conducted a genome-wide SNP screening based on the HapMap and 1000 Genomes databases for forensic individual identifications, of which the MAF values were in the range from 0.35 to 0.43 (Huang et al., 2018). Briefly, SNP loci with higher MAF values are recommended for individual identifications, which also tend to produce higher heterozygosity values. Thus, for the purpose of individual identifications, SNP loci showing relatively homogeneous frequency distributions as well as high MAF values among tested populations are recommended.

In order to determine whether these studied IISNPs have abilities of population differentiations, we delineated two PCA plots based on the population and individual levels. The population discrepancies on the PCA plots were readily discerned, particularly for African populations that were always clearly distinguished from the other four intercontinental populations. Conversely, populations from East Asia, America, South Asia, and Europe were prone to exhibit relatively close genetic relationships in comparison with the African populations based on these 93 IISNPs. This speculation is clearly verified by Figure 3B, where all the individuals other than the Africans formed inconspicuous clusters. For the NJ tree presented in Figure 3C, the Northwest Hui group was initially grouped with East Asian populations and exhibited the furthest genetic relationships from African populations. The abovementioned results indicated that the population genetic structures revealed by these 93 shared IISNPs conveyed certain disparities, which might be attributed to the uneven frequency distributions of some IISNP loci among intercontinental populations, especially between African and non-African populations.

To validate the notion mentioned above, we executed an *I*_n_ statistical analysis to evaluate the distinguishing abilities of 93 IISNPs across five intercontinental population groups. In the present study, African populations showed the largest discrepancy values at 30 of the 93 IISNP loci in comparison with the other four intercontinental population groups, in which the loci rs1335873, rs1528460, rs722098, and rs1028528 ranked in the top four with the discrepancy *I*_n_ values greater than 0.1. According to a previous report, molecular markers with high *I*_n_ values are more liable to infer population structure than those with low *I*_n_ values (Rosenberg et al., 2003). However, when the *I*_n_ statistic was utilized to screen ancestral information loci, the explicit threshold value was not clearly unified across different studies (Rosenberg et al., 2003; Xu et al., 2008; Phillips, 2015; Zeng et al., 2016; Jin et al., 2020). The recommended *I*_n_ threshold value (*I*_n_ > 0.1) for selecting ancestral markers could be acquired from a previous study (Jin et al., 2020), of which the criteria indicated that the four loci mentioned above might be suitable for ancestral information inferences. In contrast with the African populations, these 93 IISNPs provided overwhelmingly lower *I*_n_ values in the American, European, East Asian, and South Asian populations. The smaller *I*_n_ values denote that the tested genetic markers exhibit more similar allelic frequency distributions in the applied populations. In combination with the ubiquitously higher MAF values among the non-African populations in Supplementary Figure S3, we thus speculated that these IISNPs might yield a slightly better performance in the non-African populations on the purpose of individual identifications. Eventually, Figure 3B showed that the Hui individuals predominantly overlapped with East Asians; in combination with the high combined PD value of these IISNPs in the Northwest Hui group, these 93 IISNPs could perform well in identifying Northwest Hui individuals.

#### The Genetic Differentiations Assessed by Y-STRs Between the Northwest Hui and Reference Populations

To comprehensively dissect the patrilineal genetic landscape of the Hui group, we enrolled the reference population data of 17 shared Y-STRs from previously published research and the YHRD database to assess the population differentiations among the Northwest Hui group and reference populations.

A heatmap of pairwise *R*_*st*_ values among the applied 59 populations in Figure 5 illustrated that all Hui groups from Chinese different regions displayed relatively close genetic relationships with East Asian populations, as well as some Central and West Asian populations. To be more specific, the histogram of pairwise *R*_*st*_ between the Northwest Hui group and the reference populations demonstrated that the Northwest Hui group revealed the lowest genetic differentiations from the reference Hui groups, except for SCH, which was congruous with the previous study (Xie et al., 2019). It was reported that the separated distribution of SCH and the Northwest Hui group was largely attributed to the different frequency distributions of the D, E, D, G, H, J1, R1a, R1b, and R2 sub-haplogroups (Xie et al., 2019). Intriguingly, according to the histogram, relatively intimate genetic relationships might exist between the Northwest Hui group and populations from Central and West Asia, as well as several East Asian populations, like GSDX. In the light of the MDS plot, the Northwest Hui group might have close genetic relationships with some Central and West Asian populations as well as several East Asian populations, such as the Dongxiang, Kazakh, Mongolian, Arab, Turkish, and Iranian populations. From the linguistic perspective, the abovementioned Arab populations belong to the Afro-Asiatic language family; Iranian populations belong to the Indo-European language family; and Turkish, Dongxiang, Kazakh, and Mongolian populations belong to the Altaic language family. The formation of a population is always accompanied by the corresponding establishment of its language family. Although the language family may not be able to exhaustively reflect the exact formation history of a population, from cultural and historical perspectives, it can still provide certain evidence as to the eventual formation history of the Hui group. In the present study, the Northwest Hui group appertained to the Sino-Tibetan family with Chinese as the predominant language (Gladney, 2020), which was more likely to cluster with populations from the Altaic and Indo-European language families based on the Y-STR analyses. Another study also reported that Chinese Hui groups maintained several Arabic and Persian phrases in their language (Dillon, 1999).

In light of historical documents, various ancestries may have participated in the eventual formation of the Chinese Hui group. Partial Islamic adherents from Central and West Asia, such as Persians, Arabs, and Turks, were encouraged to migrate to China during several medieval dynasties, especially the Yuan (1,271–1,368 AD) Dynasty ruled by the Mongolians. It was believed that these Muslims entered China via two routes: from Northwest China via the Silk Road and from Southeastern coastal areas via the Maritime Silk Road. The Silk Road served as a series of extensive inland trade routes connecting East and West Eurasia and promoted the exchanges of economies, cultures, politics, and religions between civilizations during the 2nd century BCE to the 18th century (Elisseeff, 2000; Gan et al., 2009). It was reported that most of these Muslims, including soldiers and traders, migrated to China via the Silk Road and intermarried with local indigenous peoples, facilitating the ultimate formation of the Chinese-speaking Hui group (Elisseeff, 2000; Gan et al., 2009). The opinions on the ethnic origin of the Chinese Dongxiang group are relatively divergent according to different historians. In short, the Dongxiang was commonly described as the descendants of Mongolian troops (1,162–1,227 AD) who settled in the Hezhou region and possibly mixed with Sarts (Arab traders and Turkic-speaking city dwellers from Central Asia) (Schwarz, 1984; Wang et al., 2018). Accordingly, it is reasonable that the Northwest Hui group externalized genetically intimate relationships with the populations from Central and West Asia, as well as Dongxiang and Mongolian groups from China.

#### Ancestral Components of the Northwest Hui Group Revealed Using AISNPs

During the formation of a population, the genetic structure might be changed due to different historical events, such as migrations or intermarriages (Mellars, 2006; Duda and Jan Zrzavý, 2016), and changes in the genetic landscape of one population might distinguish it from other populations. Therefore, partial genetic variations found in modern populations can be treated as ancestral informative markers, such as AIM-STRs (Pereira et al., 2011; Phillips et al., 2011), AISNPs (Phillips et al., 2012; Kidd et al., 2014; Phillips et al., 2014; Jin et al., 2020), and AIM-InDels (Romanini et al., 2015), shedding light on the population evolutionary process.

The raw data of 26 reference populations were initially utilized to estimate the performance of 55 AISNPs in ancestral estimation and further disclose the genetic structure and ancestral bioinformation of the Northwest Hui group. As presented in Figure 7, the PCA result at the individual level indicated that individuals from East Asia, South Asia, Europe, and Africa could evidently be separated, while individuals from America were substantially overlapped with South Asians and Europeans. As depicted in a previous study, American populations displaying affinities with European populations might be largely attributed to the immigration of Europeans to the American continent since the discovery of America by Christopher Columbus in 1492 (Jordan et al., 2019). Since the 1700s, a large proportion of South Asians have immigrated to America and intermarried with indigenous Americans; consequently, over 3.4 million Americans can trace their ancestry back to South Asia according to the 2010 census (Perez and Hirschman, 2009). These historical records might partially explain why the Americans overlapped the Europeans and South Asians in the PCA plot. Other studies also reported that American populations from the 1000 Genomes Project might retain ancestral components from European, African, and indigenous American populations, which posed challenges in the ancestral analyses of American populations (Pakstis et al., 2019b; Jin et al., 2020). STRUCTURE analyses in Figure 8 supported the abovementioned opinion, in which the genetic components revealed in American populations partially coincided with those from European and South Asian populations at *K* = 3 or *K* = 4. Thus, the above results demonstrated that these 55 AISNPs could perform well in the estimation of the individual ancestral components, especially for individuals from East Asia, South Asia, Africa, and Europe.

In terms of the studied Hui group, the Hui individuals were largely assembled with East Asians in the PCA plot, demonstrating that the Northwest Hui group might retain a significant amount of genetic components from East Asia. This opinion was also congruent with the results of the STRUCTURE analyses. At *K* = 3, the studied Hui group shared the similar proportion of genetic components with East Asian populations with a large area of yellow component; a trace amount of genetic component in purple which was mainly found in European populations was also detected in the studied Hui group. We further performed ancestral component prediction of the 90 Hui individuals. In Figure 9, the Northwest Hui group demonstrated the major genetic component from East Asia and the minor component from Europe, which were roughly consistent with the genetic component distributions of the Northwest Hui group at *K* = 3 in the STRUCTURE analysis (Figure 8).

In order to have a more comprehensive genetic interpretation of the Northwest Hui group, a phylogenetic tree was further delineated based on the allelic frequency data of the Northwest Hui group and 109 reference populations. In the phylogenetic tree plot, the Northwest Hui group was initially clustered with East Asian populations, exhibiting extremely low genetic differentiations from the Tu, Xibe, Yi, Bai, Han, Hakka, and Mongolian groups, etc. The intimate genetic relationships between the Hui group and some abovementioned Chinese populations were also reported in previous studies (Hong et al., 2007; Fang et al., 2018; He et al., 2018; Lan et al., 2018; Xie et al., 2018; Wang et al., 2019; Xie et al., 2019; Chen et al., 2020; Zhou et al., 2020). For example, the close genetic relationships between Hui groups and Han populations have been commonly reported from different perspectives, including Y-STRs and Y-SNPs (Wang et al., 2019; Xie et al., 2019), STRs (Fang et al., 2018), AISNPs (He et al., 2018), InDels (Xie et al., 2018; Zhou et al., 2020), and mitochondrial DNA (Chen et al., 2020). Other populations, such as Mongolian (Hong et al., 2007) and Xibe (Lan et al., 2018), were also reported to possibly have undergone gene exchange with Hui groups to some extent.

The relatively intimate genetic relationships between the Northwest Hui and East Asian populations were subsequently certificated by two PCA plots. The genetic affinities between the Hui group and East Asian populations were further supported by previously published studies (Yao et al., 2016; He et al., 2018; Xie et al., 2018; Wang et al., 2019; Xie et al., 2019; Jin et al., 2020; Zhou et al., 2020). In detail, He et al. investigated the genetic background of the NXH group using 165 AISNPs and discovered that the genetic components of the NXH group was predominantly contributed by the East Asian ancestral component (He et al., 2018). Another study based on InDels loci also claimed that East Asian populations provided a large proportion of ancestral component for the NXH group (Zhou et al., 2020). The GSH group was also explored by researchers and was found to exhibit substantial genetic intimacy with East Asian populations (Yao et al., 2016; Xie et al., 2018; Jin et al., 2020). To be more precise, the studied Hui group might have closer genetic relationships with the reference Hui and the Tu groups. The Chinese Tu group, predominately dwelling in Northwestern China, was officially recognized as one of the 56 ethnic groups in 1953. The majority of the Tu people speak the Monguor language, which pertains to the family of Mongolic language, one of the largest sub-branches of the Altaic language family. There are different opinions on the ethnogenesis of the Tu group, and various studies indicated that its genetic origin could be traced back to Tuyuhun Xianbei, to the Mongolian troop that came to the current Qinghai-Gansu region during the Mongolian conquest, or to the Han population (Schwarz, 1984; Hu, 2010). The Chinese Hui group was once conquered by the Yuan Dynasty (Schwarz, 1984; Xie and Shan, 2002) as aforementioned, and according to previous studies, the Hui group might exchange a large proportion of genes with the Chinese Han population due to the long-term intermarriages (Schwarz, 1984; Chen et al., 2020). These historical records and genetic studies might partially explain the intimate relationship between the Northwest Hui and Tu group revealed by the PCA plot.

However, the genetic background of the Northwest Hui group estimated using AISNPs showed certain divergence from that assessed using Y-STRs. In addition to the reference Hui groups from Chinese different regions, the Northwest Hui group depicted using Y-STRs was more prone to display closer genetic relationships with populations from Central and West Asia, as well as several Chinese groups. Additionally, our previous research based on the complete mitochondrial genome illustrated that the Northwest Hui group typically exhibited closer genetic relationships with East Asian populations, roughly concordant with the genetic result provided by 55 AISNPs in this study (Chen et al., 2020). Briefly, our previous study indicated that the genetic component of the maternal lineage that appeared in the Northwest Hui group might be predominantly derived from East Asia (Chen et al., 2020); the Y chromosome strictly follows the paternal inheritance with little recombination during the genetic process, so the paternal lineage of the Northwest Hui group may still retain some ancient genetic imprints closely related to Central or West Asian populations (Gladney, 1998). Considering the ancestral component prediction, the Northwest Hui group revealed a large proportion of ancestral components from East Asia and relatively little from Europe. Thus, we speculated that a sex bias phenomenon might exist in the long-term intermarriage process of the Northwest Hui group, denoting that males from West or Central Asia might intermarry females from East Asia, and eventually facilitating the formation of the current Hui group. This perspective is also supported by previous research (Wang et al., 2019) and historical material (Schwarz, 1984).

## Conclusion

In this study, a total of 231 genetic markers were originally applied in 90 Hui male individuals dwelling in Northwest China. According to the current research, the studied genetic markers displayed satisfactory forensic performances in the Northwest Hui group, especially for the application of sequence polymorphisms which significantly enhanced the genetic diversities of STR genetic markers. Both 27 A-STRs and 94 IISNPs were polymorphic enough and could yield high forensic efficiencies in the forensic applications, such as paternity testings and individual identifications. Four of 94 IISNP loci, the rs1335873, rs1528460, rs722098, and rs1028528 loci, exhibited relatively larger discrepancies in the distributions of allelic frequencies among intercontinental populations, demonstrating that these loci might contain certain potential for the ancestry inferences. Studies based on the Y-STRs illustrated that the Northwest Hui group initially presented closest genetic relationships with reference Hui groups from Chinese different regions except for SCH and also externalized closer genetic relationships with populations from Central and West Asia, as well as several Chinese groups. Yet, the genetic structure revealed using the AISNPs indicated that the Northwest Hui group evidently displayed genetic affinities with populations from East Asia rather than those from Central or West Asia. In combination with the ancestral component estimation, it was proven that the Northwest Hui group contained a large proportion of ancestral components from East Asia and relatively little from Europe. The aforementioned results possibly indicated that the sex bias phenomenon of intermarriages might have existed in the formation of the Northwest Hui group, involving more intermarriages between males from Central and West Asia and females from East Asia.

## Data Availability

The data is available in NCBI PRJNA738655.

## References

[B1] Al-jumaah R. Musthafa M. M. Al-Shaikh M. Badri O. M., (2012). J. o. B. Hussein 11, 16539–16545.

[B2] Alexander D. H. Novembre J. Lange K. (2009). Fast Model-Based Estimation of Ancestry in Unrelated Individuals. Genome Res. 19, 1655–1664. 10.1101/gr.094052.109 19648217 PMC2752134

[B3] Almalki N. Chow H. Y. Sharma V. Hart K. Siegel D. Wurmbach E. (2017). Systematic Assessment of the Performance of Illumina's MiSeq FGx Forensic Genomics System. ELECTROPHORESIS 38, 846–854. 10.1002/elps.201600511 27943350

[B4] Araújo A. M. d. Guimarães S. E. F. Machado T. M. M. Lopes P. S. Pereira C. S. Silva F. L. R. d. (2006). Genetic Diversity between Herds of Alpine and Saanen Dairy Goats and the Naturalized Brazilian Moxotó Breed. Genet. Mol. Biol. 29, 67–74. 10.1590/s1415-47572006000100014

[B5] Auton A. Abecasis G. R. Altshuler D. M. Durbin R. M. Abecasis G. R. Bentley D. R. (2015). Nature 526, 68–74. 26432245

[B6] Balloux F. Lugon-Moulin N. (2002). The Estimation of Population Differentiation with Microsatellite Markers. Mol. Ecol. 11, 55–65. 10.1046/j.0962-1083.2001.01436.x 11856418

[B7] Børsting C. Morling N. (2015). Next Generation Sequencing and its Applications in Forensic Genetics. Forensic Sci. Int. Genet. 18, 78–89. 10.1016/j.fsigen.2015.02.002 25704953

[B8] Botstein D. White R. L. Skolnick M. Davis R. W. (1980). Construction of a Genetic Linkage Map in Man Using Restriction Fragment Length Polymorphisms. Am. J. Hum. Genet. 32, 314–331. 6247908 PMC1686077

[B9] Bruijns B. Tiggelaar R. Gardeniers H. (2018). Massively Parallel Sequencing Techniques for Forensics: A Review. Electrophoresis 39, 2642–2654. 10.1002/elps.201800082 30101986 PMC6282972

[B10] Chang C. C. Chow C. C. Tellier L. C. Vattikuti S. Purcell S. M. Lee J. J. (2015). Second-generation PLINK: Rising to the challenge of Larger and Richer Datasets. GigaSci. 4, 7. 10.1186/s13742-015-0047-8 PMC4342193 25722852

[B11] Chen C. Li Y. Tao R. Jin X. Guo Y. Cui W. (2020). The Genetic Structure of Chinese Hui Ethnic Group Revealed by Complete Mitochondrial Genome Analyses Using Massively Parallel Sequencing. Genes 11, 1352. 10.3390/genes11111352 PMC7698084 33202591

[B12] Churchill J. D. Novroski N. M. M. King J. L. Seah L. H. Budowle B. (2017). Population and Performance Analyses of Four Major Populations with Illumina's FGx Forensic Genomics System. Forensic Sci. Int. Genet. 30, 81–92. 10.1016/j.fsigen.2017.06.004 28651097

[B13] Churchill J. D. Schmedes S. E. King J. L. Budowle B. (2016). Evaluation of the Illumina Beta Version ForenSeq DNA Signature Prep Kit for Use in Genetic Profiling. Forensic Sci. Int. Genet. 20, 20–29. 10.1016/j.fsigen.2015.09.009 26433485

[B104] Cifuentes L. O. Martínez E. H. Acuña M. P. Jonquera H. G. (2006). Probability of Exclusion in Paternity Testing: Time to Reassess. J. Forensic Sci. 51 (2), 349–350. 10.1111/j.1556-4029.2006.00064.x 16566769

[B14] Dávila S. G. Gil M. G. Resino-Talaván P. Campo J. L. (2009). Evaluation of Diversity between Different Spanish Chicken Breeds, a Tester Line, and a White Leghorn Population Based on Microsatellite Markers. Poult. Sci. 88, 2518–2525. 10.3382/ps.2009-00347 19903949

[B15] Delest A. Godfrin D. Chantrel Y. Ulus A. Vannier J. Faivre M. (2020). Sequenced-based French Population Data from 169 Unrelated Individuals with Verogen's ForenSeq DNA Signature Prep Kit. Forensic Sci. Int. Genet. 47, 102304. 10.1016/j.fsigen.2020.102304 32417726

[B17] Dillon M. (1999). China's Muslim Hui Community: Migration, Settlement and Sects. London: Curzon Press.

[B18] Duda P. Jan Zrzavý Z. (2016). Human Population History Revealed by a Supertree Approach. Sci. Rep. 6, 29890. 10.1038/srep29890 27431856 PMC4949479

[B19] Elisseeff V. (2000). The Silk Roads : Highways of Culture and Commerce.

[B20] England R. Nancollis G. Stacey J. Sarman A. Min J. Harbison S. (2020). Compatibility of the ForenSeq DNA Signature Prep Kit with Laser Microdissected Cells: An Exploration of Issues that Arise with Samples Containing Low Cell Numbers. Forensic Sci. Int. Genet. 47, 102278. 10.1016/j.fsigen.2020.102278 32413702

[B21] Fan H. Du Z. Wang F. Wang X. Wen S.-Q. Wang L. (2020). The Forensic Landscape and the Population Genetic Analyses of Hainan Li Based on Massively Parallel Sequencing DNA Profiling. bioRxiv. 10.1101/2020.03.27.011064 33847803

[B22] Fang Y. Guo Y. Xie T. Jin X. Lan Q. Zhou Y. (2018). Forensic Molecular Genetic Diversity Analysis of Chinese Hui Ethnic Group Based on a Novel STR Panel. Int. J. Leg. Med. 132, 1297–1299. 10.1007/s00414-018-1829-1 29582135

[B23] Fattorini P. Previderé C. Carboni I. Marrubini G. Sorçaburu-Cigliero S. Grignani P. (2017). Performance of the ForenSeqTMDNA Signature Prep Kit on Highly Degraded Samples. Electrophoresis 38, 1163–1174. 10.1002/elps.201600290 28078776

[B24] Fisher R. A. (1951). Standard Calculations for Evaluating a Blood-Group System. Heredity 5, 95–102. 10.1038/hdy.1951.5 14840760

[B25] Fullwood M. J. Wei C.-L. Liu E. T. Ruan Y. (2009). Next-generation DNA Sequencing of Paired-End Tags (PET) for Transcriptome and Genome Analyses. Genome Res. 19, 521–532. 10.1101/gr.074906.107 19339662 PMC3807531

[B26] Gan F. Brill R. H. Tian S. (2009). Ancient Glass Research along the Silk Road. World Scientific.

[B27] Gettings K. B. Aponte R. A. Vallone P. M. Butler J. M. (2015). STR Allele Sequence Variation: Current Knowledge and Future Issues. Forensic Sci. Int. Genet. 18, 118–130. 10.1016/j.fsigen.2015.06.005 26197946

[B28] Gettings K. B. Kiesler K. M. Faith S. A. Montano E. Baker C. H. Young B. A. (2016). Sequence Variation of 22 Autosomal STR Loci Detected by Next Generation Sequencing. Forensic Sci. Int. Genet. 21, 15–21. 10.1016/j.fsigen.2015.11.005 26701720 PMC5093907

[B29] Gladney D. C. (1998). Ethnic Identity in China: The Making of a Muslim Minority Nationality. Harcourt Brace College Publishers.

[B30] Gladney D. C. (2020). Muslim Chinese: Ethnic Nationalism in the People’s Republic. Netherlands: Brill.

[B31] Gouy A. Zieger M. (2017). STRAF-A Convenient Online Tool for STR Data Evaluation in Forensic Genetics. Forensic Sci. Int. Genet. 30, 148–151. 10.1016/j.fsigen.2017.07.007 28743032

[B32] Guo F. (2017). Population Genetics for 17 Y-STR Loci in Hui Ethnic Minority from Liaoning Province, Northeast China. Forensic Sci. Int. Genet. 28, e36–e37. 10.1016/j.fsigen.2017.02.011 28256335

[B33] Guo F. Yu J. Zhang L. Li J. (2017). Massively Parallel Sequencing of Forensic STRs and SNPs Using the Illumina ForenSeq DNA Signature Prep Kit on the MiSeq FGx Forensic Genomics System. Forensic Sci. Int. Genet. 31, 135–148. 10.1016/j.fsigen.2017.09.003 28938154

[B34] Guo H. Yan J. Jiao Z. Tang H. Zhang Q. Zhao L. (2008). Genetic Polymorphisms for 17 Y-Chromosomal STRs Haplotypes in Chinese Hui Population. Leg. Med. 10, 163–169. 10.1016/j.legalmed.2007.11.001 18187357

[B35] He G. Wang Z. Wang M. Luo T. Liu J. Zhou Y. (2018). Forensic Ancestry Analysis in Two Chinese Minority Populations Using Massively Parallel Sequencing of 165 Ancestry-Informative SNPs. Electrophoresis 39, 2732–2742. 10.1002/elps.201800019 29869338

[B36] Hollard C. Ausset L. Chantrel Y. Jullien S. Clot M. Faivre M. (2019). Automation and Developmental Validation of the ForenSeq DNA Signature Preparation Kit for High-Throughput Analysis in Forensic Laboratories. Forensic Sci. Int. Genet. 40, 37–45. 10.1016/j.fsigen.2019.01.010 30739830

[B37] Hong W. Chen S. Shao H. Fu Y. Hu Z. Xu A. (2007). HLA Class I Polymorphism in Mongolian and Hui Ethnic Groups from Northern China. Hum. Immunol. 68, 439–448. 10.1016/j.humimm.2007.01.020 17462512

[B38] Hu A. J. (2010). An Overview of the History and Culture of the Xianbei (‘Monguor'/‘Tu'). Asian Ethn. 11, 95–164. 10.1080/14631360903531958

[B39] Huang E. Liu C. Zheng J. Han X. Du W. Huang Y. (2018). Genome-wide Screen for Universal Individual Identification SNPs Based on the HapMap and 1000 Genomes Databases. Sci. Rep. 8, 5553. 10.1038/s41598-018-23888-0 29615764 PMC5882920

[B40] Hussing C. Bytyci R. Huber C. Morling N. Børsting C. (2019). The Danish STR Sequence Database: Duplicate Typing of 363 Danes with the ForenSeq DNA Signature Prep Kit. Int. J. Leg. Med. 133, 325–334. 10.1007/s00414-018-1854-0 29797283

[B41] Illumina (2015). Forenseq™ Universal Analysis Software Guide, Document #15053876v01. Available at: https://verogen.com/wp-content/uploads/2018/08/ForenSeq-Univ-Analysis-SW-Guide-VD2018007-A.pdf .

[B43] Jäger A. C. Alvarez M. L. Davis C. P. Guzmán E. Han Y. Way L. (2017). Developmental Validation of the MiSeq FGx Forensic Genomics System for Targeted Next Generation Sequencing in Forensic DNA Casework and Database Laboratories. Forensic Sci. Int. Genet. 28, 52–70. 10.1016/j.fsigen.2017.01.011 28171784

[B44] Jin X. Y. Guo Y. X. Chen C. Cui W. Liu Y. F. Tai Y. C. (2020). Ancestry Prediction Comparisons of Different AISNPs for Five Continental Populations and Population Structure Dissection of the Xinjiang Hui Group Via a Self-Developed Panel. Genes (Basel), 11 (5), 505. 10.3390/genes11050505 PMC7288656 32375366

[B45] Jordan I. K. Rishishwar L. Conley A. B. (2019). Native American Admixture Recapitulates Population-specific Migration and Settlement of the continental United States. Plos Genet. 15, e1008225. 10.1371/journal.pgen.1008225 31545791 PMC6756731

[B46] Just R. S. Moreno L. I. Smerick J. B. Irwin J. A. (2017). Performance and Concordance of the ForenSeq System for Autosomal and Y Chromosome Short Tandem Repeat Sequencing of Reference-type Specimens. Forensic Sci. Int. Genet. 28, 1–9. 10.1016/j.fsigen.2017.01.001 28126691

[B47] Khubrani Y. M. Hallast P. Jobling M. A. Wetton J. H. (2019). Massively Parallel Sequencing of Autosomal STRs and Identity-Informative SNPs Highlights Consanguinity in Saudi Arabia. Forensic Sci. Int. Genet. 43, 102164. 10.1016/j.fsigen.2019.102164 31585345

[B48] Kidd K. K. Pakstis A. J. Speed W. C. Grigorenko E. L. Kajuna S. L. B. Karoma N. J. (2006). Developing a SNP Panel for Forensic Identification of Individuals. Forensic Sci. Int. 164, 20–32. 10.1016/j.forsciint.2005.11.017 16360294

[B49] Kidd K. K. Speed W. C. Pakstis A. J. Furtado M. R. Fang R. Madbouly A. (2014). Progress toward an Efficient Panel of SNPs for Ancestry Inference. Forensic Sci. Int. Genet. 10, 23–32. 10.1016/j.fsigen.2014.01.002 24508742

[B50] Köcher S. Müller P. Berger B. Bodner M. Parson W. Roewer L. (2018). Inter-laboratory Validation Study of the ForenSeq DNA Signature Prep Kit. Forensic Sci. Int. Genet. 36, 77–85. 10.1016/j.fsigen.2018.05.007 29945120

[B51] Lan Q. Chen J. Guo Y. Xie T. Fang Y. Jin X. (2018). Genetic Structure and Polymorphism Analysis of Xinjiang Hui Ethnic Minority Based on 21 STRs. Mol. Biol. Rep. 45, 99–108. 10.1007/s11033-018-4143-6 29372494

[B52] Li H. Zhao X. Ma K. Cao Y. Zhou H. Ping Y. (2017). Applying Massively Parallel Sequencing to Paternity Testing on the Ion Torrent Personal Genome Machine. Forensic Sci. Int. Genet. 31, 155–159. 10.1016/j.fsigen.2017.09.007 28946114

[B74] Li R. Li H. Peng D. Hao B. Wang Z. Huang E. 2018, 38.

[B53] Liu Y. Wen S. Guo L. Bai R. Shi M. Li X. (2018). Haplotype Data of 27 Y-STRs Analyzed in the Hui and Tujia Ethnic Minorities from China. Forensic Sci. Int. Genet. 35, e7–e9. 10.1016/j.fsigen.2018.04.006 29685746

[B54] Liu Y. Yu T. Mei S. Jin X. Lan Q. Zhou Y. (2020). Forensic Characteristics and Genetic Affinity Analyses of Xinjiang Mongolian Group Using a Novel Six Fluorescent Dye-Labeled Typing System Including 41 Y-STRs and 3 Y-InDels. Mol. Genet. Genomic Med. 8, e1097. 10.1002/mgg3.1097 31876394 PMC7005640

[B98] Liu Y. Yang J. Li Y. Tang R. Yuan D. Wang Y. (2021). 12.

[B55] Ma X. Sun R. Hao C. (2017). Polymorphism of 15 Short Tandem Repeat Loci in Hui Population of Ningxia Tongxin District. J. forensic Leg. Med. 52, 168–171. 10.1016/j.jflm.2017.08.014 28942264

[B56] Malaspinas A.-S. Slatkin M. Song Y. S. (2011). Match Probabilities in a Finite, Subdivided Population. Theor. Popul. Biol. 79, 55–63. 10.1016/j.tpb.2011.01.003 21266180 PMC3065509

[B57] Marshall T. C. Slate J. Kruuk L. E. B. Pemberton J. M. (1998). Statistical Confidence for Likelihood‐based Paternity Inference in Natural Populations. Mol. Ecol. 7, 639–655. 10.1046/j.1365-294x.1998.00374.x 9633105

[B58] Mellars P. (2006). Going East: New Genetic and Archaeological Perspectives on the Modern Human Colonization of Eurasia. Science 313, 796–800. 10.1126/science.1128402 16902130

[B59] Meng H.-T. Han J.-T. Zhang Y.-D. Liu W.-J. Wang T.-J. Yan J.-W. (2014). Diversity Study of 12 X-Chromosomal STR Loci in Hui Ethnic from China. Electrophoresis 35, 2001–2007. 10.1002/elps.201400045 24723364

[B60] Nei M. (1973). Analysis of Gene Diversity in Subdivided Populations. Proc. Natl. Acad. Sci. 70, 3321–3323. 10.1073/pnas.70.12.3321 4519626 PMC427228

[B61] Nei M. (1987). Molecular Evolutionary Genetics. Columbia University Press.

[B62] Novroski N. M. M. King J. L. Churchill J. D. Seah L. H. Budowle B. (2016). Characterization of Genetic Sequence Variation of 58 STR Loci in Four Major Population Groups. Forensic Sci. Int. Genet. 25, 214–226. 10.1016/j.fsigen.2016.09.007 27697609

[B63] Pakstis A. J. Gurkan C. Dogan M. Balkaya H. E. Dogan S. Neophytou P. I. (2019). Genetic Relationships of European, Mediterranean, and SW Asian Populations Using a Panel of 55 AISNPs. Eur. J. Hum. Genet. 27, 1885–1893. 10.1038/s41431-019-0466-6 31285530 PMC6871633

[B64] Pakstis A. J. Haigh E. Cherni L. ElGaaied A. B. A. Barton A. Evsanaa B. (2015). 52 Additional Reference Population Samples for the 55 AISNP Panel. Forensic Sci. Int. Genet. 19, 269–271. 10.1016/j.fsigen.2015.08.003 26355664

[B65] Pakstis A. J. Kang L. Liu L. Zhang Z. Jin T. Grigorenko E. L. (2017). Increasing the Reference Populations for the 55 AISNP Panel: the Need and Benefits. Int. J. Leg. Med. 131, 913–917. 10.1007/s00414-016-1524-z PMC5491587 28070634

[B66] Pakstis A. J. Speed W. C. Soundararajan U. Rajeevan H. Kidd J. R. Li H. (2019). Population Relationships Based on 170 Ancestry SNPs from the Combined Kidd and Seldin Panels. Sci. Rep. 9, 18874. 10.1038/s41598-019-55175-x 31827153 PMC6906462

[B67] Parson W. Ballard D. Budowle B. Butler J. M. Gettings K. B. Gill P. (2016). Massively Parallel Sequencing of Forensic STRs: Considerations of the DNA Commission of the International Society for Forensic Genetics (ISFG) on Minimal Nomenclature Requirements. Forensic Sci. Int. Genet. 22, 54–63. 10.1016/j.fsigen.2016.01.009 26844919

[B68] Pereira L. Alshamali F. Andreassen R. Ballard R. Chantratita W. Cho N. S. (2011). PopAffiliator: Online Calculator for Individual Affiliation to a Major Population Group Based on 17 Autosomal Short Tandem Repeat Genotype Profile. Int. J. Leg. Med. 125, 629–636. 10.1007/s00414-010-0472-2 20552217

[B69] Perez A. D. Hirschman C. (2009). The Changing Racial and Ethnic Composition of the US Population: Emerging American Identities. C. Hirschman 35, 1–51. 10.1111/j.1728-4457.2009.00260.x PMC2882688 20539823

[B70] Phillips C. Fernandez-Formoso L. Garcia-Magariños M. Porras L. Tvedebrink T. Amigo J. (2011). Analysis of Global Variability in 15 Established and 5 New European Standard Set (ESS) STRs Using the CEPH Human Genome Diversity Panel. Forensic Sci. Int. Genet. 5, 155–169. 10.1016/j.fsigen.2010.02.003 20457091

[B71] Phillips C. Fondevila M. Lareau M. V. (2012). A 34-plex Autosomal SNP Single Base Extension Assay for Ancestry Investigations. Methods Mol. Biol. (Clifton, N.J.) 830, 109–126. 10.1007/978-1-61779-461-2_8 22139656

[B72] Phillips C. (2015). Forensic Genetic Analysis of Bio-Geographical Ancestry. Forensic Sci. Int. Genet. 18, 49–65. 10.1016/j.fsigen.2015.05.012 26013312

[B73] Phillips C. Parson W. Lundsberg B. Santos C. Freire-Aradas A. Torres M. (2014). Building a Forensic Ancestry Panel from the Ground up: The EUROFORGEN Global AIM-SNP Set. Forensic Sci. Int. Genet. 11, 13–25. 10.1016/j.fsigen.2014.02.012 24631693

[B75] Romanini C. Romero M. Salado Puerto M. Catelli L. Phillips C. Pereira R. (2015). Ancestry Informative Markers: Inference of Ancestry in Aged Bone Samples Using an Autosomal AIM-Indel Multiplex. Forensic Sci. Int. Genet. 16, 58–63. 10.1016/j.fsigen.2014.11.025 25531060

[B76] Rosenberg N. A. Li L. M. Ward R. Pritchard J. K. (2003). Informativeness of Genetic Markers for Inference of Ancestry*. Am. J. Hum. Genet. 73, 1402–1422. 10.1086/380416 14631557 PMC1180403

[B77] Sanger F. Nicklen S. Coulson A. R. (1977). DNA Sequencing with Chain-Terminating Inhibitors. Proc. Natl. Acad. Sci. 74, 5463–5467. 10.1073/pnas.74.12.5463 271968 PMC431765

[B78] Schwarz H. G. (1984). The Minorities Of Northern China: A Survey, Center for East Asian Studies. Bellingham, WA: Western Washington University, East Asian Studies Press, Vol. 8.

[B79] Sharma V. Jani K. Khosla P. Butler E. Siegel D. Wurmbach E. (2019). Evaluation of ForenSeq™ Signature Prep Kit B on Predicting Eye and Hair Coloration as Well as Biogeographical Ancestry by Using Universal Analysis Software (UAS) and Available Web-Tools. Electrophoresis 40, 1353–1364. 10.1002/elps.201800344 30767247

[B80] Sharma V. van der Plaat D. A. Liu Y. Wurmbach E. (2020). Analyzing Degraded DNA and Challenging Samples Using the ForenSeq DNA Signature Prep Kit. Sci. Justice 60, 243–252. 10.1016/j.scijus.2019.11.004 32381241

[B81] Sheriff O. Alemayehu K. (2018). Genetic Diversity Studies Using Microsatellite Markers and Their Contribution in Supporting Sustainable Sheep Breeding Programs: A Review. Cogent Food Agric. 4, 1459062. 10.1080/23311932.2018.1459062

[B82] Shete S. Tiwari H. Elston R. C. (2000). On Estimating the Heterozygosity and Polymorphism Information Content Value. Theor. Popul. Biol. 57, 265–271. 10.1006/tpbi.2000.1452 10828218

[B83] Silvia A. L. Shugarts N. Smith J. (2017). A Preliminary Assessment of the ForenSeq FGx System: Next Generation Sequencing of an STR and SNP Multiplex. Int. J. Leg. Med. 131, 73–86. 10.1007/s00414-016-1457-6 27785563

[B84] Tamura K. Stecher G. Peterson D. Filipski A. Kumar S. (2013). MEGA6: Molecular Evolutionary Genetics Analysis Version 6.0. Mol. Biol. Evol. 30, 2725–2729. 10.1093/molbev/mst197 24132122 PMC3840312

[B85] Team R. C. (2016). A Language and Environment for Statistical Computing. Vienna, Austria: R Foundation for Statistical Computing.

[B86] Tillmar A. (2010). Populations and Statistics in Forensic Genetics [Internet]. PhD dissertation. Sweden: Linköping University Electronic Press, Linköping University Medical Dissertations. Available at: http://urn.kb.se/resolve?urn=urn:nbn:se:liu:diva-54742 .

[B87] Vandeputte M. (2012). An Accurate Formula to Calculate Exclusion Power of Marker Sets in Parentage Assignment. Genet. Sel. Evol. 44, 36. 10.1186/1297-9686-44-36 23206351 PMC3523974

[B88] Wang C. C. Lu Y. Kang L. Ding H. Yan S. Guo J. (2019). The Massive Assimilation of Indigenous East Asian Populations in the Origin of Muslim Hui People Inferred from Paternal Y Chromosome. Am. J. Phys. Anthropol. 169, 341–347. 10.1002/ajpa.23823 30889274

[B89] Wang J. Wen S. Shi M. Liu Y. Zhang J. Bai R. (2018). Haplotype Structure of 27 YfilerPlus Loci in Chinese Dongxiang Ethnic Group and its Genetic Relationships with Other Populations. Forensic Sci. Int. Genet. 33, e13–e16. 10.1016/j.fsigen.2017.12.014 29402655

[B90] Wendt F. R. Churchill J. D. Novroski N. M. M. King J. L. Ng J. Oldt R. F. (2016). Genetic Analysis of the Yavapai Native Americans from West-Central Arizona Using the Illumina MiSeq FGx Forensic Genomics System. Forensic Sci. Int. Genet. 24, 18–23. 10.1016/j.fsigen.2016.05.008 27243782

[B91] Xavier C. Parson W. (2017). Evaluation of the Illumina ForenSeq DNA Signature Prep Kit - MPS Forensic Application for the MiSeq FGx Benchtop Sequencer. Forensic Sci. Int. Genet. 28, 188–194. 10.1016/j.fsigen.2017.02.018 28279935

[B92] Xie M. Song F. Li J. Lang M. Luo H. Wang Z. (2019). Genetic Substructure and Forensic Characteristics of Chinese Hui Populations Using 157 Y-SNPs and 27 Y-STRs. Forensic Sci. Int. Genet. 41, 11–18. 10.1016/j.fsigen.2019.03.022 30927697

[B93] Xie T. Guo Y. Chen L. Fang Y. Tai Y. Zhou Y. (2018). A Set of Autosomal Multiple InDel Markers for Forensic Application and Population Genetic Analysis in the Chinese Xinjiang Hui Group. Forensic Sci. Int. Genet. 35, 1–8. 10.1016/j.fsigen.2018.03.007 29602069

[B94] Xie T. Shen C. Jin X. Lan Q. Fang Y. Zhu B. (2020). Genetic Structural Differentiation Analyses of Intercontinental Populations and Ancestry Inference of the Chinese Hui Group Based on a Novel Developed Autosomal AIM-InDel Genotyping System. Biomed. Research International 2020, 2124370. 10.1155/2020/2124370 32908873 PMC7468629

[B95] Xie X. Shan X. (2002). Research on the Hui.

[B96] Xu S. Huang W. Qian J. Jin L. (2008). Analysis of Genomic Admixture in Uyghur and its Implication in Mapping Strategy. Am. J. Hum. Genet. 82, 883–894. 10.1016/j.ajhg.2008.01.017 18355773 PMC2427216

[B97] Yao H.-B. Wang C.-C. Tao X. Shang L. Wen S.-Q. Zhu B. (2016). Genetic Evidence for an East Asian Origin of Chinese Muslim Populations Dongxiang and Hui. Sci. Rep. 6, 38656. 10.1038/srep38656 27924949 PMC5141421

[B99] Yousefi S. Abbassi-Daloii T. Kraaijenbrink T. Vermaat M. Mei H. van ‘t Hof P. (2018). A SNP Panel for Identification of DNA and RNA Specimens. BMC Genomics 19, 90. 10.1186/s12864-018-4482-7. 29370748 PMC5785835

[B100] Zeng X. Chakraborty R. King J. L. Larue B. Moura-Neto R. S. Budowle B. (2016). Selection of Highly Informative SNP Markers for Population Affiliation of Major US Populations. Int. J. Leg. Med. 130, 341–352. 10.1007/s00414-015-1297-9 26645290

[B101] Zhou B. Wen S. Sun H. Zhang H. Shi R. (2020). Genetic Affinity between Ningxia Hui and Eastern Asian Populations Revealed by a Set of InDel Loci. R. Soc. Open Sci. 7, 190358. 10.1098/rsos.190358 32218926 PMC7029925

[B102] Zhu B.-F. Zhang Y.-D. Liu W.-J. Meng H.-T. Yuan G.-L. Lv Z. (2014). Genetic Diversity and Haplotype Structure of 24 Y-Chromosomal STR in Chinese Hui Ethnic Group and its Genetic Relationships with Other Populations. Electrophoresis 35, 1993–2000. 10.1002/elps.201300574 24789806

[B103] Zou X. Wang Z. He G. Wang M. Liu J. Wang S. (2020). Genetic Variation and Population Structure Analysis of Chinese Wuzhong Hui Population Using 30 Indels. Ann. Hum. Biol. 47, 300–303. 10.1080/03014460.2020.1736627 32202169

