# Supplementary figures and images for: Comprehensive Insights Into Forensic Features and Genetic Background of Chinese Northwest Hui Group Using Six Distinct Categories of 231 Molecular Markers
Source: Front Genet. 2021 Oct 15;12:705753. doi: 10.3389/fgene.2021.705753 (PMC8555763; doi:10.3389/fgene.2021.705753)

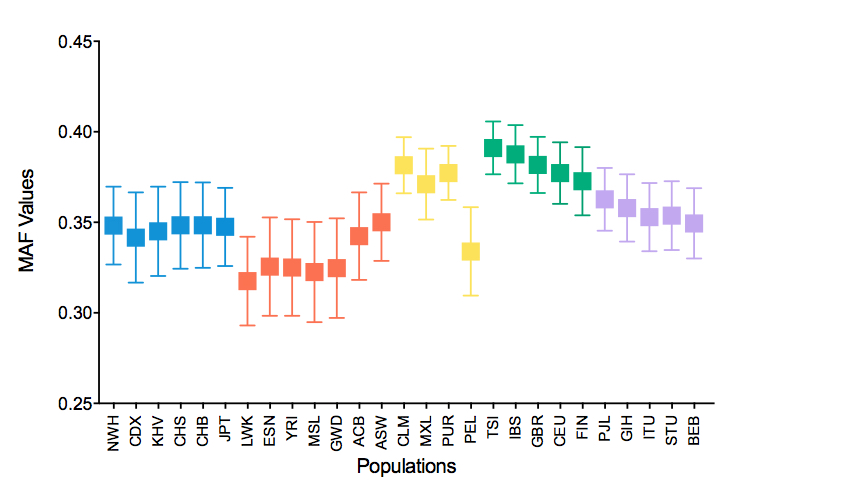

Supplement: Supplementary file 2 [file Image3.JPEG]

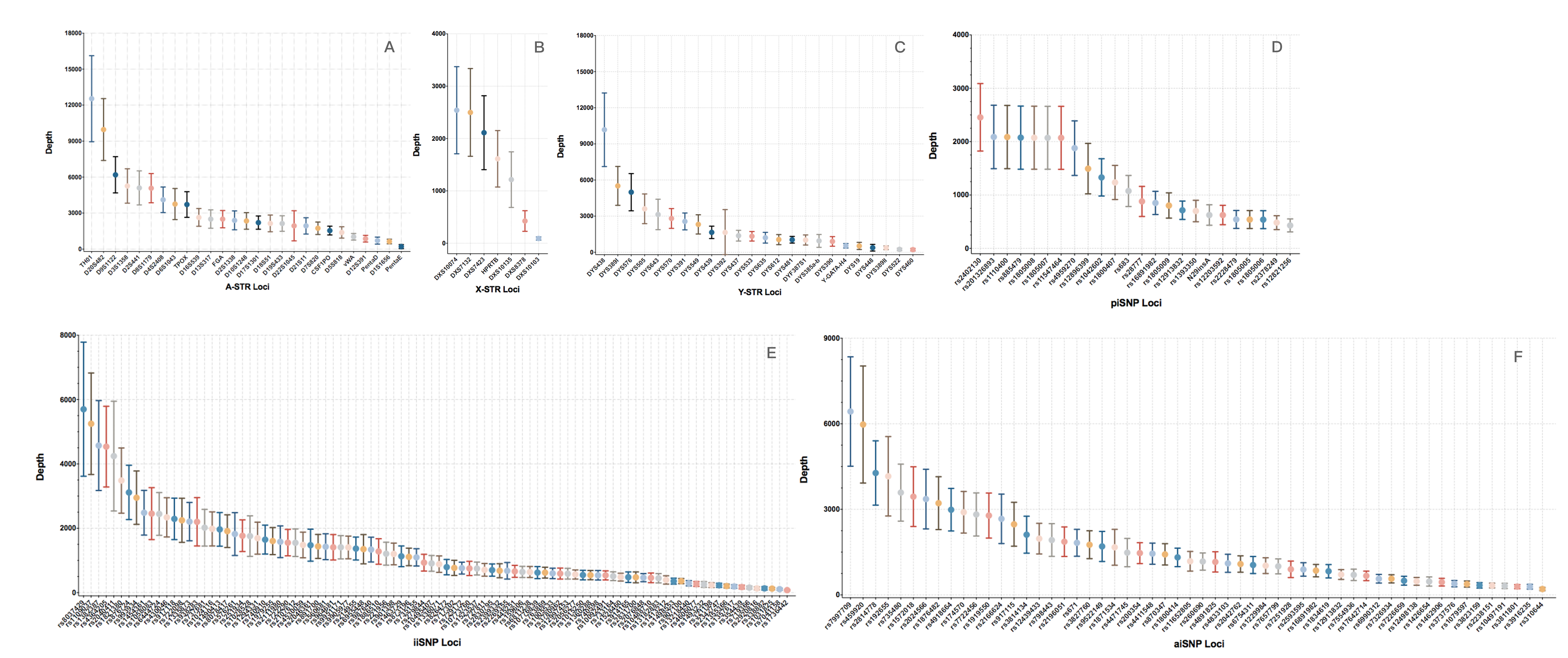

Supplement: Supplementary file 5 [file Image1.JPEG]

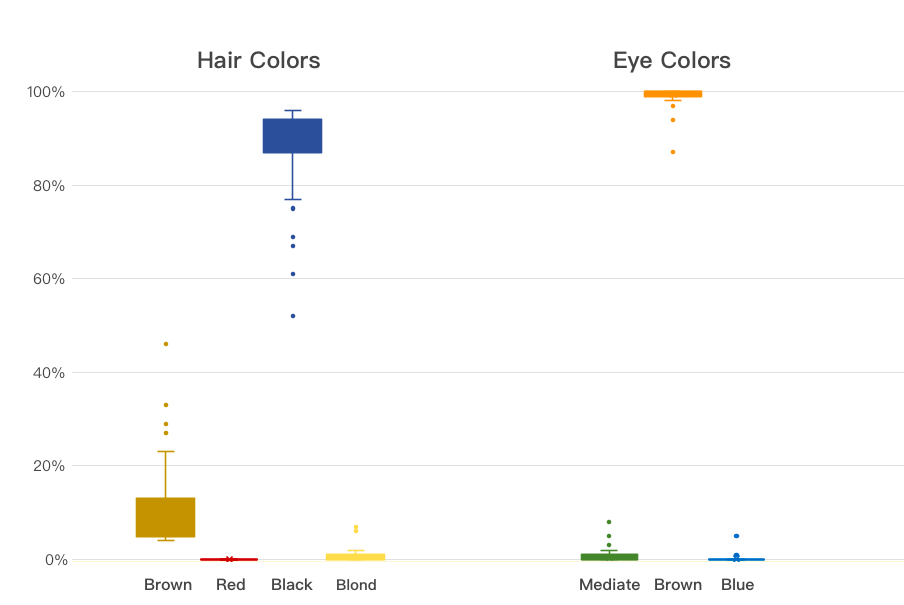

Supplement: Supplementary file 6 [file Image2.JPEG]
